# Bis-Cinnamamide Derivatives as APE/Ref-1 Inhibitors for the Treatment of Human Melanoma

**DOI:** 10.3390/molecules27092672

**Published:** 2022-04-21

**Authors:** Razan Alhazmi, Shirley Tong, Shaban Darwish, Elina Khanjani, Bharti Khungar, Swati Chawla, Zhonghui Zheng, Richard Chamberlin, Keykavous Parang, Sun Yang

**Affiliations:** 1 Center for Targeted Drug Delivery, Department of Biomedical and Pharmaceutical Sciences, Chapman University School of Pharmacy, Irvine, CA 92618, USA; alhaz101@mail.chapman.edu (R.A.); fong149@mail.chapman.edu (S.T.); shaban_darwish@yahoo.com.sg (S.D.); bsharifi@chapman.edu (E.K.); bkhungar@pilani.bits-pilani.ac.in (B.K.); chawlaswati@hotmail.com (S.C.); 2 Department of Pharmaceutical Sciences, University of California Irvine, Irvine, CA 92697, USA; zhengzhonghui@sina.com (Z.Z.); richard.chamberlin@uci.edu (R.C.); 3 Department of Pharmacy Practice, Chapman University School of Pharmacy, Irvine, CA 92618, USA

**Keywords:** APE/Ref-1, human melanoma, small molecular inhibitors, reactive oxygen species (ROS), redox regulation

## Abstract

Human malignant melanoma exhibits imbalances in redox status, leading to activation of many redox-sensitive signaling pathways. APE/Ref-1 is a multifunctional protein that serves as a redox chaperone that regulates many nuclear transcription factors and is an important mechanism in cancer cell survival of oxidative stress. Previous studies showed that APE/Ref-1 is a potential druggable target for melanoma therapy. In this study, we synthesized a novel APE/Ref-1 inhibitor, bis-cinnamoyl-1,12-dodecamethylenediamine (**2**). In a xenograft mouse model, compound **2** treatment (5 mg/kg) significantly inhibited tumor growth compared to the control group, with no significant systemic toxicity observed. We further synthesized compound **2** analogs to determine the structure-activity relationship based on their anti-melanoma activities. Among those, 4-hydroxyphenyl derivative (**11**) exhibited potent anti-melanoma activities and improved water solubility compared to its parental compound **2**. The IC_50_ of compound **11** was found to be less than 0.1 μM. Compared to other known APE/Ref-1 inhibitors, compound **11** exhibited increased potency in inhibiting melanoma proliferation. As determined by luciferase reporter analyses, compound **2** was shown to effectively inhibit H_2_O_2_-activated AP-1 transcription activities. Targeting APE/Ref-1-mediated signaling using pharmaceutical inhibitors is a novel and effective strategy for melanoma treatment with potentially high impact.

## 1. Introduction

Melanoma arising from melanocytes makes up only 2% of all skin cancers, while it causes over 80% of all skin cancer deaths [1]. In recent years, melanoma incidence rates have increased more rapidly than other cancer types in the United States, and it is expected that 7650 patients will die due to melanoma in 2022 alone [2]. Ultraviolet radiation (UVR,) which can generate reactive oxygen species (ROS) and nitric oxide (NO) in the skin [3,4,5], has been implicated as a significant environmental contributor to the development of melanoma [6,7]. The contributions of UVR to melanomagenesis and malignant development may, at least partially, be due to ROS- or NO-induced DNA damage, which has been studied in our laboratory and by other groups [8,9,10,11]. 

APE/Ref-1 is a critical enzyme in DNA base excision repair, which catalyzes the cleavage of the phosphodiester bond at either the 5′ or 3′ end of an apurinic/apyrimidinic site [12]. Studies have also revealed that APE-1 exhibits distinct redox functions that facilitate DNA binding activities of many nuclear transcription factors (such as AP-1, Myb, NF-κB, p53, and HIF-α); due to its redox-regulatory activity, it is also known as Redox Factor-1 (Ref-1) [13]. APE/Ref-1 plays a critical role in malignant transformation when combined with elevated ROS levels in the JB6 model [14]. Notably, our previous studies demonstrated that APE/Ref-1 is induced by ROS and NO stress [8,15]. In addition, genome-wide analyses and proteomic studies also suggest an important role of APE/Ref-1 in regulating many biological processes, such as mitochondrial function and ribosomal RNA quality control [16].

Accumulating evidence shows that many human tumors, including the brain, cervical, and prostate cancers, exhibit elevated APE/Ref-1 expression and abnormal subcellular localization compared to normal tissues, making it an attractive druggable target for developing new cancer therapeutic strategies [12,17,18]. Up-regulated APE/Ref-1 is shown to protect cells from various pro-apoptosis stimuli, including oxidative stresses, chemotherapeutic drugs, and radiation treatment [19,20]. Elevated APE/Ref-1 has been closely associated with increased metastasis of cancer cells and contributes to tumor development and progression in human melanoma and hepatocellular carcinoma [15,21,22]. Compounds impairing APE/Ref-1-mediated redox signaling or DNA repair have shown enhanced anti-tumor activities [23,24,25]; however, other studies have also demonstrated the pro-apoptotic effects of APE/Ref-1 by potentiating p53-mediated signaling [26]. The experimental stresses applied in the studies may explain the distinct role of APE/Ref-1 in apoptosis. 

In addition, given the complexity of cancer signaling networks, there are wide varieties of intra- and intercellular signaling pathways that could fuel cancer progression [27]. APE/Ref-1 regulates many nuclear transcription factors in a redox-dependent and -independent manner. Inhibition of APE/Ref-1 may generate broader inhibition of its regulated signaling network, compared to blocking a single signaling pathway downstream of APE/Ref-1 [12,28]. Direct inhibition may be more efficient and minimize the development of drug resistance.

**Figure 1:**
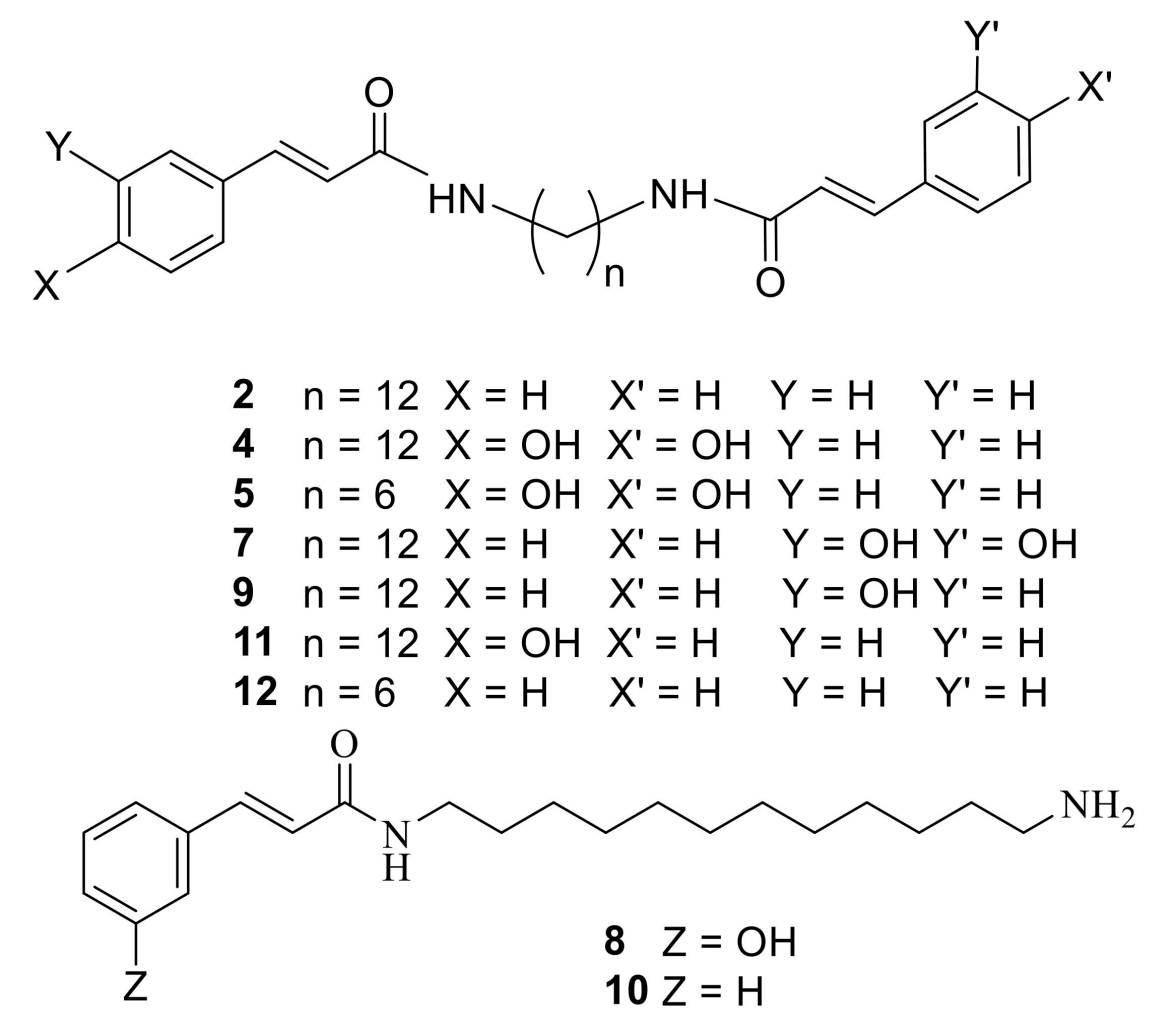
Chemical structures of cinnamamide derivatives.

The crystal structure of APE/Ref-1 revealed potentially druggable pockets in the vicinity of Cys65, which is located in the redox regulation domain. The studies showed that redox-specific inhibition of APE/Ref-1 induced cell cycle arrest and sensitized cancer cells to chemotherapy [28,29,30,31,32]. We have conducted virtual screenings using the ICM software package developed by Molsoft [33,34,35], which docks small molecules from a large database and ranks binding affinity using an energetic scoring function. The chemical structures of the top hit compounds were identified [15], and a series of analogs based on the lead compound structure were designed and synthesized in our study. Among them, bis-cinnamoyl-1,12-dodecamethylenediamine (compound **2**) (Figure 1) exhibited the most potent anti-melanoma activity both *in vitro* and *in vivo*. The structure-activity relationship generated from our study will shed light on the future design and synthesis of small molecular inhibitors targeting APE/Ref-1 for melanoma treatment.

## 2. Results

### 2.1. Chemistry

**Scheme 1:**
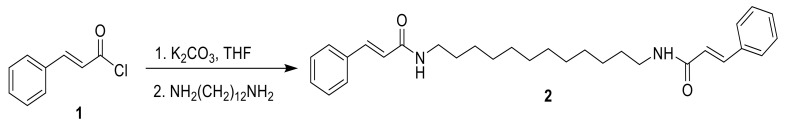
Synthesis of bis-cinnamoyl-1,12-dodecamethylenediamine (**2**).

**Scheme 2:**
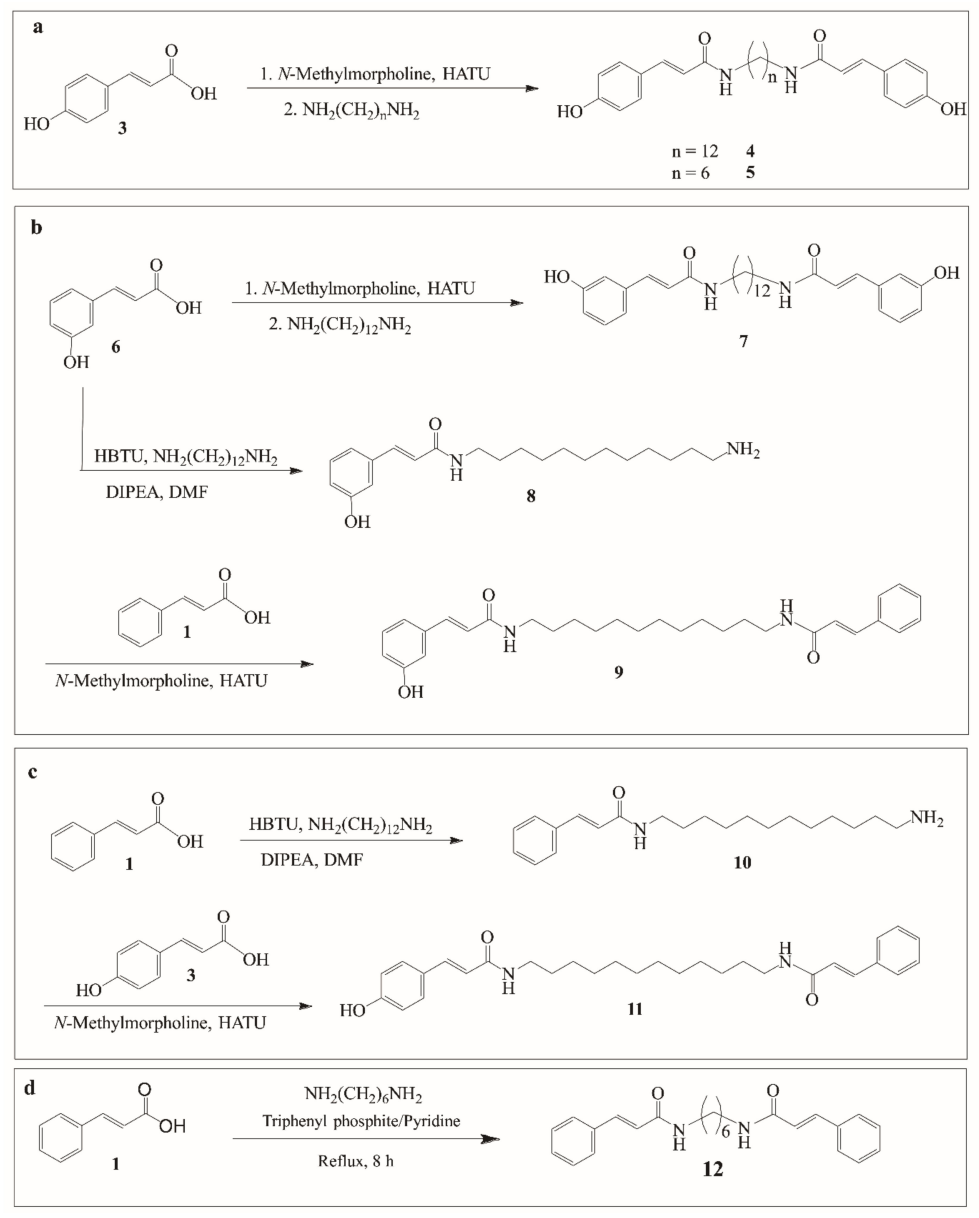
Synthesis of bis-cinnamoyl derivatives. (**a**) Synthesis of symmetrical bis-cinnamoyl derivatives **4** and **5**; (**b**) Synthesis of symmetrical bis-cinnamoyl derivative **7** and unsymmetrical bis-cinnamoyl derivative **9**; (**c**) Synthesis of unsymmetrical bis-cinnamoyl derivative **11**; (**d**) Synthesis of symmetrical bis-cinnamoyl derivative **12**.

In this study, a series of symmetrical and unsymmetrical dicinnamoyl derivatives conjugated through hydrophobic central units of 1,12-diaminododecane and 1,6-diaminohexane were synthesized as depicted in Scheme 1 and Scheme 2. Different bolaamphiphiles were prepared based on increasing the hydrophilicity of these compounds by introducing hydrophilic hydroxyl groups to the aromatic rings and changing the hydrophobic chain length. 

First, bis-cinnamoyl-1,12-dodecamethylenediamine (**2**) was synthesized by the reacting cinnamoyl chloride (**1**) with 1,12-dodecamethylenediamine in dry THF in the presence of potassium carbonate as a base (Scheme 1). The structure of the product was confirmed by NMR and mass spectrometry. The purity was confirmed by analytical HPLC, showing a retention time (R_t_) of 26 min.

To improve water solubility, diamide bolaamphiphiles containing two hydroxyl groups were prepared using *N*-methylmorpholine (NMM) and 1-[bis(dimethylamino)methylene]-1H-1,2,3-triazolo[4,5-b]pyridinium 3-oxide hexafluorophosphate (Hexafluorophosphate Azabenzotriazole Tetramethyl Uronium, HATU) as a coupling reagent. Symmetrical bolaamphiphile derivatives (**4**, **5**, and **7**) were prepared through a coupling reaction of one equivalent of 1,12-dodecamethylenediamine with two equivalents of the cinnamic acid derivative under basic conditions; 4-Hydroxycinnamic acid (**3**) and 3-hydroxycinnamic acid (**6**) were activated by HATU, followed by a coupling reaction with 1,12-dodecamethylenediamine in the presence of NMM as a basic catalyst to generate the corresponding diamide, bis-(4-hydroxy-cinnamoyl)-1,12-dodecamethylenediamine (**4**) and bis-(3-hydroxy-cinnamoyl)-1,12-dodecamethylenediamine (**7**), respectively (Scheme 2a,b). Analytical HPLC confirmed the purity of compounds **4** and **7**, showing (R_t_) = 21.89 and 22.19 min, respectively; moreover, to prepare a more hydrophilic bolaamphiphile derivative, a shorter aliphatic 1,6-dihexylamine was used for the reaction with compound **3** to prepare bis-(4-hydroxy-cinnamoyl)-1,6-hexanemethylenediamine (**5**) under the same conditions, as mentioned above (Scheme 2a). The crude products were purified by silica gel column chromatography and the purity was confirmed using analytical HPLC (Supplementary Materials).

Unsymmetrical bis-cinnamamide derivatives **9** and **11** were synthesized in two steps. First, the reaction of equimolar amounts of the appropriate acid derivative and the diamine was used to afford the corresponding monocinnamamide. It should be noted that optimizing the reaction conditions, such as using a mild activating reagent and reaction time control, was required to drive the reaction towards a single coupling and create the desired compound containing a free amine group for the subsequent coupling. As depicted in Scheme 2b,c, *N*-(12-aminododecyl)-3-(3-hydroxyphenyl) acrylamide (**8**) and *N*-(12-aminododecyl)-cinnamamide (**10**) were constructed from the reaction of 3-hydroxycinnamic acid (**6**) and cinnamic acid (**1**), respectively, with diamine in the presence of HBTU and DIPEA over 2 h. As a representative example, the novel monoamide (**8**) was identified via mass spectrometry, exhibiting a mass of 347.1 [M + H]^+^; moreover, the ^1^H NMR spectra showed broad singlet and triplet peaks at 7.69 and 8.08 ppm, which are characteristics of NH_2_ and NH, respectively. The singlet peak at 9.57 ppm represents the phenolic hydroxyl group (Figure S13, Supplementary Materials).

Second, using similar conditions as used in the synthesis of compound **4**, monocinnamoyl-fatty acyl amide conjugates (**8** and **10**) containing free amino groups were reacted with the additional cinnamic acid derivatives (**1** and **3**), respectively. As shown in Scheme 2b,c, these reactions afforded the corresponding unsymmetrical bis-cinnamoyl derivatives, *N*-(12-cinnamamidododecyl)-3-(3-hydroxyphenyl) acrylamide (**9**) and *N*-(12-cinnamamidododecyl)-3-(4-hydroxyphenyl) acrylamide (**11**), with each possessing only one hydroxyl group. As a representative example, the ^1^H NMR spectrum fits well with structure **9** and indicated that the molecule has unsymmetrical imide protons, showing two triplet signals at 7.92 and 8.08 ppm.

Using an alternative method, as shown in Scheme 2d, hexane-1,6-diamine was reacted with cinnamic acid in the presence of pyridine and triphenyl phosphite for 8 h to afford (2E,2’E)-*N*,*N*’-(Propane-1,3-diyl)bis(3-phenylacrylamide) (**12**).

### 2.2. Molecular Docking Study of the Synthesized Compound Candidates

**Figure 2:**
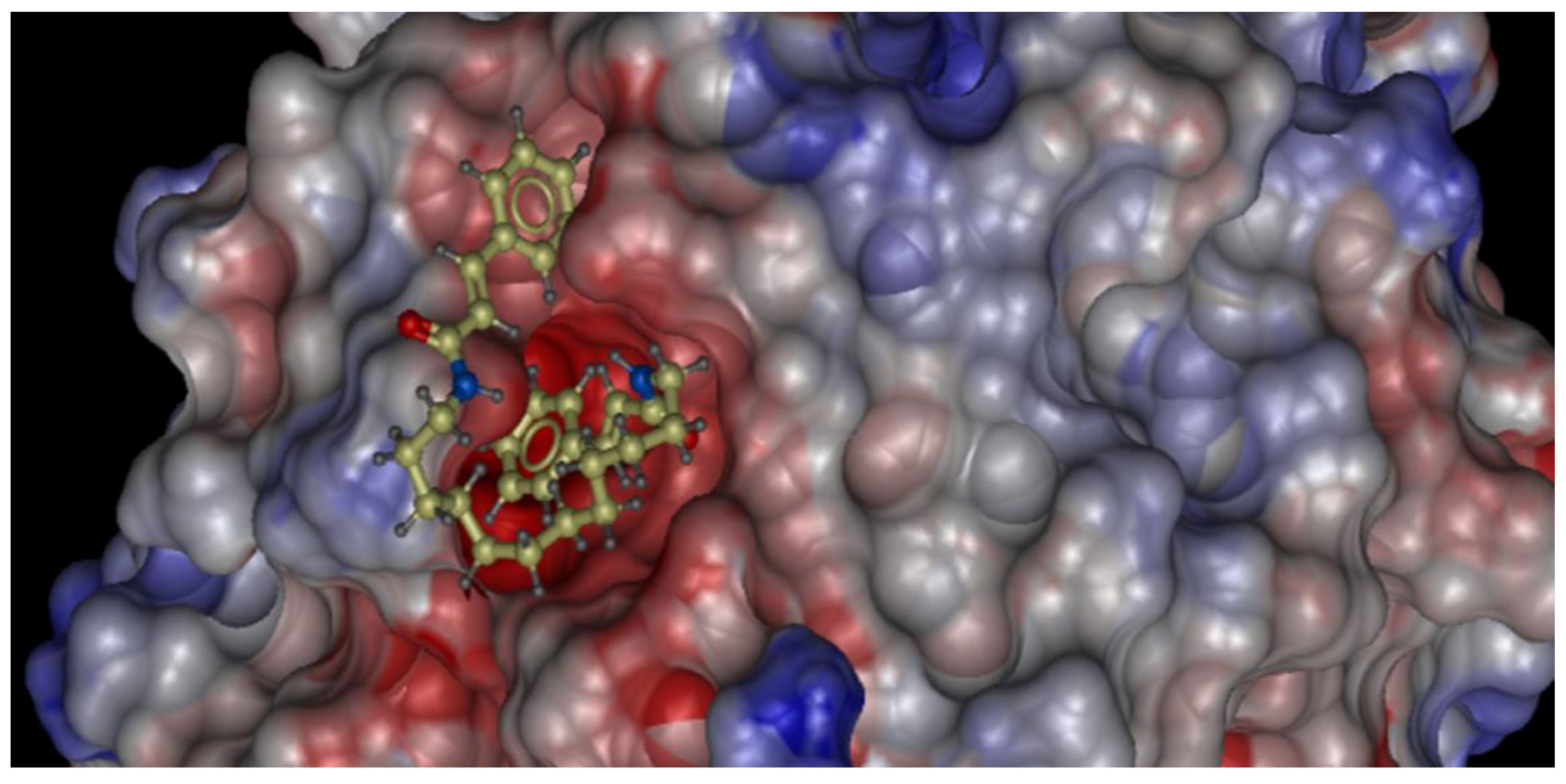
Molecular docking and binding mode of compound **2** to the druggable binding site of the APE/Ref-1 protein (1BIX).

Using molecular modeling, we further evaluated the new compounds by docking the compounds to the APE/Ref-1 crystal structure, downloaded from PDB (1BIX) (Table 1) [15]. Figure 2 shows the binding of compound **2** into its binding site of APE/Ref-1, resulting in an ICM docking score of −9.23. Compound **11** docked into the same binding cavity, with an ICM docking score of −16.31. The best docking score among all the synthesized candidate compounds was observed with Compound **4** (−20.14), as shown in Table 1.

### 2.3. Effects of Novel APE/Ref-1 Inhibitors on Melanoma Proliferation Using MTT Colorimetric Assay

APE/Ref-1 inhibitors have exhibited anti-proliferation and anti-angiogenesis activity against several human malignancies; therefore, we have chosen anti-tumor efficacy as an endpoint to rank the candidate compounds for bio-evaluation. MTT assays were conducted to evaluate the in vitro anti-melanoma activities of the synthesized compounds in human melanoma cell lines and immortalized melanocytes. Cells were incubated with compounds at various concentrations for 72 h. Cell viabilities after treatments were then analyzed in comparison to that of control.

**Figure 3:**
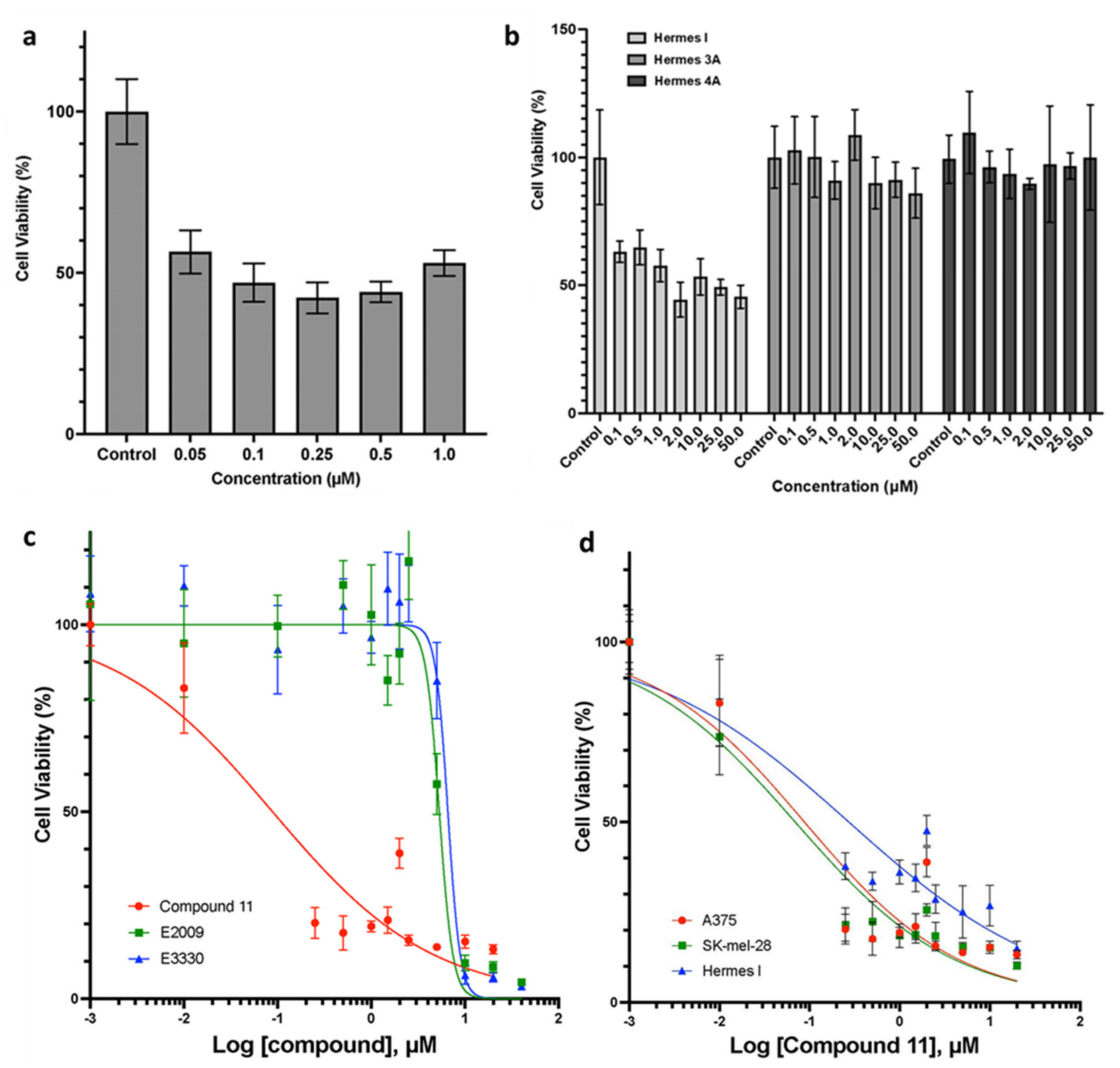
Potent cytotoxicity of compounds **2** and **11** in human melanoma cells. Cell viability was determined by MTT assay. The results are shown as the percentage of viable cells after 72 h treatment at different concentrations. The results were statistically processed from three independent experiments. The IC_50_ was calculated using GraphPad Prism 8. (**a**) Cytotoxicity of compound **2** in A375 melanoma cells; (**b**) Cytotoxicity of compound **2** in human immortalized melanoblasts cells (Hermes 1, 3A, and 4A). The bars represent the average of three independent experiments. Hermes 1, Hermes 3A, and Hermes 4A cell lines were obtained from the Wellcome Trust Functional Genomics Cell Bank and cultured as instructed [37,38]. (**c**) Cytotoxicity of compound **11** in comparison to APE/Ref−1 inhibitors E2009 and E3330 in A375 melanoma cells. (**d**) Melanoma cells are more sensitive to compound **11** in comparison to melanoblast Hermes 1 cells.

As shown in Figure 3a, lead compound **2** exhibited potent cytotoxicity in human melanoma A375 cells at 0.1 µM as the surviving cells were reduced to less than 50% of control after 72 h treatment; however, with increasing doses, the cellular toxicity was not increased accordingly in both cell lines, which may be explained by the limited water solubility and stability of compound **2** (Figure 3a). Apparent drug precipitation was evident in the media at higher concentrations (≥2.5 µM). Of note, no significant cytotoxicity of compound **2** was observed among two melanoblast cell lines after 72 h treatment (Hermes 3A and Hermes 4A, Figure 3b). Hermes 1 cells were more sensitive to compound **2**, but the cytotoxicity was not elevated with increasing concentrations.

Compounds **4** and **7** are analogs of lead compound **2** in which two hydrophilic hydroxyl groups (−OH) were added to the aromatic rings. The virtual docking scores of compounds **4** and **7** analyzed by the ICM-Pro program are −20.14 and −11.74, respectively. Compared to compound **2** (−9.23), compound **4** exhibited improved docking to the druggable pocket localized in the redox domain of APE/Ref-1; however, the MTT results showed these modifications led to a significant loss of anti-tumor activity compared to compound **2**.

Similar to compound **4**, compound **5** has two hydrophilic groups (−OH) but includes a modified chain length to increase the water solubility and was found to have a virtual docking score of −12.91. MTT analysis demonstrated that such modification also resulted in the loss of anti-melanoma activity compared to compound **2**, and 50% cell death was not observed up to 50 µM (Table 1).

Compounds **9** and **11** are analogs of lead compound **2**, in which one hydrophilic hydroxyl group (−OH) was added to the *meta-* or *para-*position, respectively. The corresponding virtual docking scores of compounds **9** and **11** are −5.97 and −16.31 (Table 1). Compound **9** showed poor anti-melanoma activity, while compound **11** exhibited a potent cytotoxic effect in melanoma cells (Figure 3). The virtual docking profiles align well with their anti-melanoma activities, suggesting the position of the −OH group plays a vital role in their interactions with the APE/Ref−1 protein. The binding profile model may guide future modifications of the aromatic ring and predict the compounds’ anti-melanoma activities; moreover, compound **11** exhibits improved water solubility compared to compound **2** due to the hydrophilic hydroxyl group. Significant precipitation was evident at ~20 µM, approximately 8-folds higher than compound **2** (2.5 µM, data not shown).

### 2.4. Potent and Selective Anti-Tumor Activities of Compound **11** in Human Melanoma Cells

Among all the synthesized analogs of compound **2,** compound **11** exhibited promising anti-tumor activities with improved water solubility in a dose-dependent manner. The viabilities of melanoma cells were significantly decreased with compound **11** treatment (Figure 3c,d). In comparison to well-studied APE/Ref-1 inhibitors, E2009 and E3330 [30,31,36], compound **11** exhibited significantly increased potency in reducing viability in melanoma cells (Figure 3c). The IC_50_s of compound **11** in A375 and SK-Mel-28 melanoma cells were 0.088 µM and 0.07 µM, respectively (Figure 3d, Table 1), which are more potent than commercially available APE/Ref-1 redox inhibitors. At 1 µM, cell viability reduced to 19% of control (vehicle only, 0.1% DMSO) after treatment with compound **11**, while no significant cytotoxicity was observed with E2009 and E3330 treatment at the same concentration (96.7% and 102.6% of control, respectively) (Figure 3c). The IC_50s_ of E3330 and E2009 in A375 melanoma cells were 6.6 µM and 5.3 µM, respectively, which were 75-fold and 60-fold higher compared to that of compound **11**.

The selectivity of compound **11** was also determined by comparing its cytotoxicity in melanoma cells to that in normal melanoblast Hermes 1 cells. As shown in Figure 3d, the IC_50_ of compound **11** in Hermes 1 is 0.274 µM, which is much higher than the IC_50s_ observed in A375 and SK-Mel-28 melanoma cells.

### 2.5. Compound 2 Suppressed H_2_O_2_-Induced AP-1 Transactivation in Human Melanoma Cells

**Figure 4:**
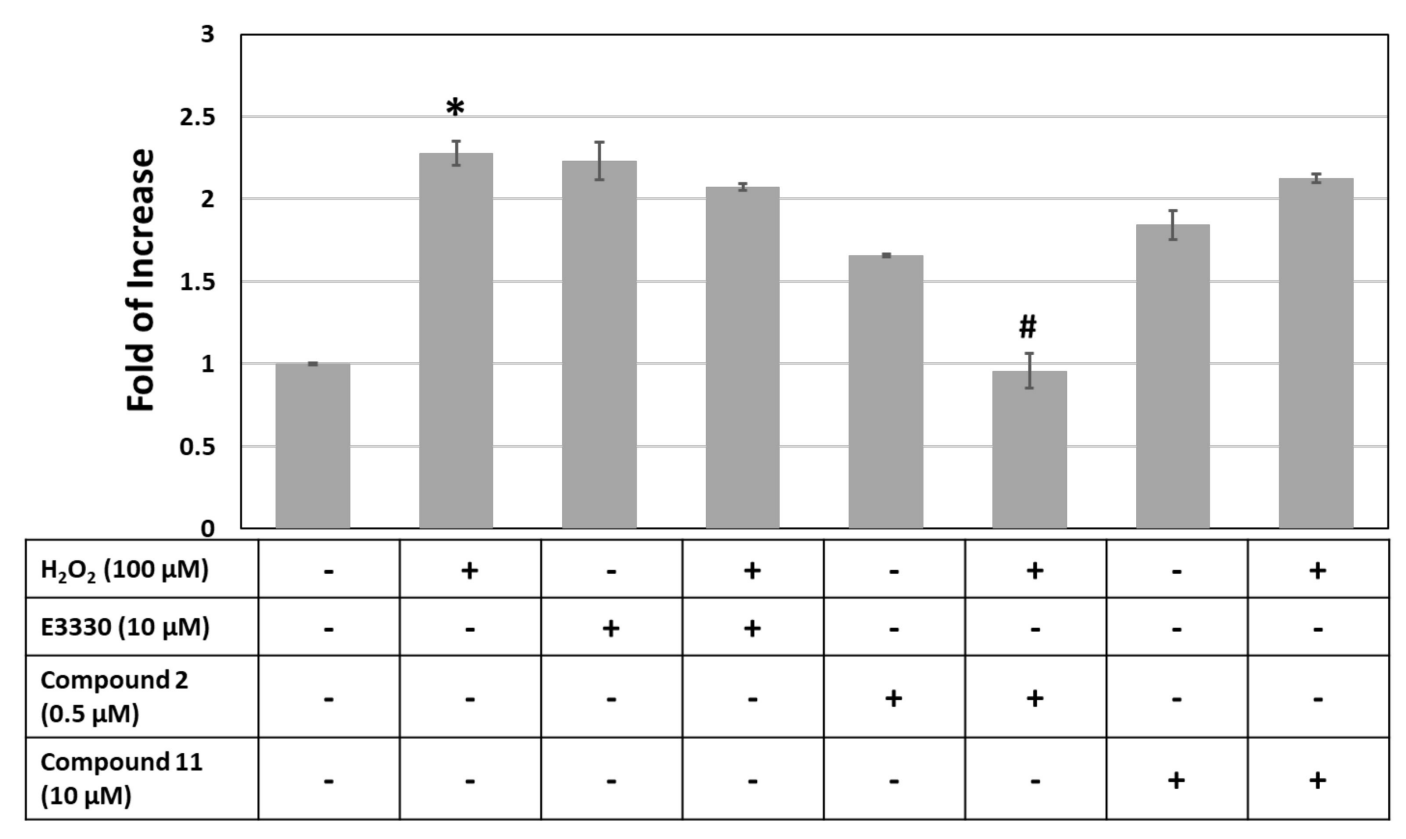
Inhibition of AP-1 transcriptional activation in human melanoma A375 cells by compound **2**. A375 cells were transfected with an AP-1 luciferase reporter plasmid. 24 h after transfection, cells were incubated with H_2_O_2_ (100 µM) in the presence or absence of compound **2** (0.5 µM), compound **11** (10 µM), or E3330 (10 µM) for 48 h. After treatment, the cells were harvested to determine the luciferase activity, expressed as fold of control without treatment. The results normalized by cell numbers represent means ± SD of triplicates. * *p* < 0.05 compared to control; # *p* < 0.05 compared to H_2_O_2_ treatment.

An AP-1 transactivation assay was conducted in melanoma cells transfected with a luciferase reporter construct as described previously [39]. Our study showed that compound **2** at 0.5 μM effectively inhibited AP-1 transactivation induced by H_2_O_2_ in A375 melanoma cells compared with H_2_O_2_ alone (*p* < 0.05) (Figure 4). The H_2_O_2_-activated AP-1 activity was reduced from 2.3-fold of control to approximately basal levels (95.7% of control); however, compound **2** did not display any inhibition of the basal AP-1-dependent transcription. Co-treatment with E3330, an APE/Ref-1 redox inhibitor, at 10 µM concentration, reduced AP-1 activity marginally. Compound **11** at 10 µM also failed to inhibit H_2_O_2_-activated AP-1 transactivation (*p* > 0.05).

### 2.6. Effects of APE/Ref-1 Inhibitor on Tumor Growth Using a Melanoma Xenograft Mouse Model

Given the limited water solubility, lead compound **2** (stocked in DMSO) was further diluted in normal saline. The drug suspension was sonicated at room temperature for 15 min before injection to nude mice. HPLC analysis shows no evidence of chemical structural change after compounding. The drug suspension was made fresh daily and injected into mice immediately after compounding. The control group was intraperitoneally injected (IP) with vehicle (0.1% DMSO in normal saline) daily based on the body weight (0.1 mL/10 g).

**Figure 5:**
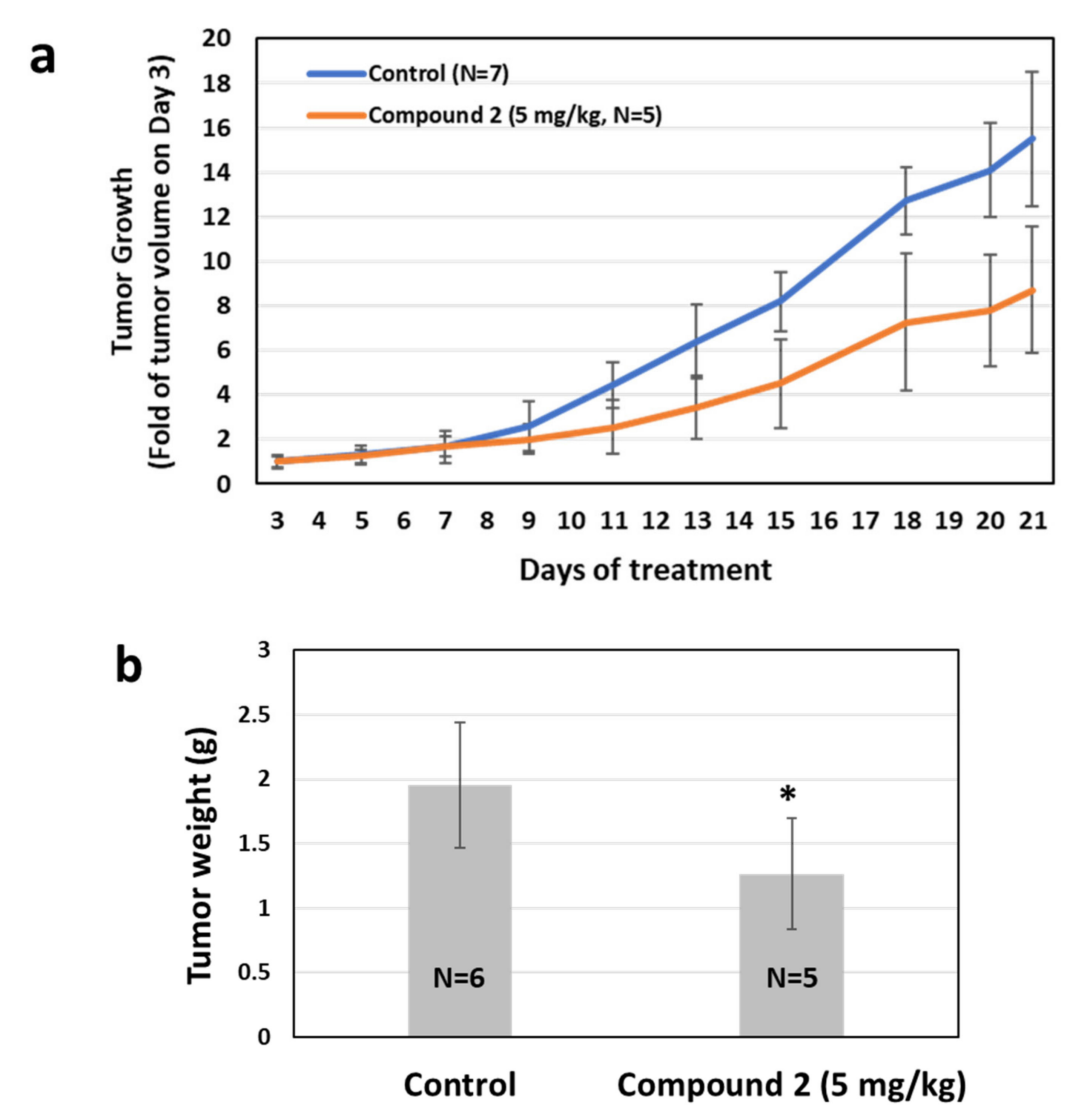
Compound **2** inhibited the tumor growth of human melanoma *in vivo*. Metastatic melanoma A375 cells were injected into male nude mice subcutaneously on the flank. Compound **2** treatment (5 mg/kg, IP, daily) inhibited tumor growth (**a**) and reduced tumor weight (**b**). (**a**) Tumor volume was measured by digital calipers and determined using the formula (mm^3^) = (L × W^2^)/2. Tumor growth was presented as a fold increase in size from day 3 (mean ± SD). (**b**) Comparison of measured tumor burden (via weight) after 21 days of therapy. The *p* values were determined using a two-tailed Student’s *t*-test. * *p* < 0.05 compared to control group.

As shown in Figure 5, the study demonstrated that the APE/Ref-1 inhibitor, compound **2,** effectively inhibited tumor growth *in vivo*. The effective dose of compound **2** was as low as 5 mg/kg IP daily without producing any apparent systemic toxicities (Figure S27, Supplementary Materials). After 21-days of treatment, tumor growth was significantly inhibited, with the average tumor size reduced to 58.5% of control (*p* < 0.05) (Figure 5a). The average tumor weight in the treatment group was only 64.7% of that of the control group (*p* < 0.05) (Figure 5b). Future studies are warranted to determine the dose-response and pharmacokinetic profile.

## 3. Discussion

In recent years, there have been dramatic developments in the treatment of advanced cutaneous melanoma (CM) using revolutionary immunotherapy, which is a powerful new approach yielding remarkable and durable responses in melanoma patients [40,41]; however, these checkpoint inhibitors are mainly indicated for patients with metastatic melanoma and disease progression following other treatments [42]. In addition, current and novel approaches have not slowed the worldwide melanoma epidemic, and the incidence of CM continues to rise in the United States [2]. As such, the development of novel therapeutic interventions to block melanomagenesis and disease progression to advanced stages will have both high impact and importance.

It has been well studied that APE/Ref-1 serves as an important mechanism facilitating cancer cell survival from oxidative stresses associated with radiation therapy and chemotherapy [15,20,43]. Novel APE/Ref-1 inhibitors have been shown to inhibit cancer cell metastasis and the development of drug resistance, suggesting that targeting APE/Ref-1 is a promising strategy to achieve a better clinical benefit for cancer patients [17,22,30,44].

In recent years, increasing efforts have been devoted to developing novel redox inhibitors of APE/Ref-1 for cancer treatment; however, to the best of our knowledge, only a limited number of compounds exhibit promising APE/Ref-1 inhibitory effects [24,30,43,45,46]. A recently developed small molecular inhibitor of apurinic/apyrimidinic (AP) endonuclease activity was shown to exacerbate the oxidative DNA damage following exposure to cisplatin at 10 µM [24,25]. To date, the most successful APE/Ref-1 redox inhibitors reported are E3330 (APX3330) and its analogs, which exhibited therapeutic effects on tumor angiogenesis and growth without interfering with its DNA repair activities [36,47]. E3330 was also shown to reduce the collective cell migration of human breast cancer cells and decrease chemo-invasion and colony formation when combined with docetaxel; however, such inhibitory activity was only evident at a higher concentration (50 µM) [30]. A recent Phase I trial of E3330 reported that six of nineteen cancer patients had disease stabilization for ≥4 cycles after treatment [23,28]. One melanoma patient presented with a stable disease with 245 days of treatment.

Beyond these studies, there has been little work on structure-based inhibitor design. As detailed in our previous studies [15,17], the redox function of APE/Ref-1 plays a vital role in melanoma progression, which is also consistent with other groups’ observation of the anti-tumor activity of APE/Ref-1 redox inhibitors. We, therefore, selected the structure of the redox domain of APE/Ref-1 for screening and molecular modeling. In our study, we carried out virtual screenings using the ICM-Pro software package developed by MolSoft L.L.C [33,34]. The program uses an energetic scoring function to carry out virtual docking of molecules from a large database and ranks binding. Notably, the software incorporates ligand flexibility. We screened multiple drug libraries containing over 3-million compounds and identified the top-ranked candidate compounds. After chemical structure optimization and cell-based bioactivity screening, we have successfully developed our lead APE/Ref-1 inhibitor, compound **2**, for further structure-activity studies.

More analogs of compound **2** were synthesized to develop the structure-activity relationship and improve the physicochemical properties. Our study demonstrated that incorporating one or two hydroxyl groups on the aromatic ring enhances the water solubility, but this comes at the expense of reducing anti-melanoma activity. Compound **11** was found to be more water-soluble than compound **2** and exhibited promising anti-melanoma activities compared to the reported APE-Ref-1 inhibitors, E3330 and E2009. Inhibition of cell viability was evident even at very low concentrations in vitro (<0.1 µM). Our in vivo mouse study showed that compound **2** treatment significantly inhibited tumor growth in a melanoma xenograft mouse model.

However, given the hydrophobic nature of compound **2**, its water solubility is limited, and increased precipitation was evident at concentrations above 2.5 µM. Shortening the chain from C12 to C6 markedly improved the compound’s water solubility, but significantly reduced the anti-melanoma activity. One hypothesis is that the long-chain may contribute to binding by facilitating or stabilizing the interaction between the compound and the APE/Ref-1 protein. Shortening the chain may lead to an unstable binding profile and subsequent reduction of anti-tumor activity. This hypothesis may apply to compound **12** as well, which possesses a shortened chain and loss of anti-melanoma activity. To improve the bioavailability and drug delivery to melanoma cells, we will also consider using a cyclic peptide drug delivery system containing arginine and tryptophan residues developed by the Parang group [48]. These cell-penetrating peptides were shown to effectively enhance the uptake of anti-cancer drugs in tumor cells [49].

Structure-based virtual screening and docking have been widely used for drug discovery, such as in hit identification and lead optimization. As noted in Table 1, the virtual docking score of a candidate compound does not always align with its anti-melanoma activities. The poor water solubility of compound **2** significantly hindered the detection of direct interactions between compound **2** and the APE/Ref-1 protein using Surface Plasmon Resonance (SPR) analysis. Future crystallization studies of APE/Ref-1 protein-ligand complexes are warranted to optimize and refine the virtual modeling and ultimately improve structure-based drug design.

## 4. Materials and Methods

### 4.1. Materials

Trans-cinnamoyl chloride, trans-4-hydroxycinnamic acid, trans-cinnamic acid, 1,6-hexamethylenediamine, 1,12-dodecamethylenediamine, all solvents, chemicals reagents, and HPLC-eluents were purchased from Sigma-Aldrich, St. Louis, MO, USA, and used as received without further purification. Analytical HPLC was used to confirm the purity of final products (≥95%). The analytical HPLC was conducted on the Shimadzu RP-HPLC system and C18 column (250 cm × 4.60 mm) over 80 min using water (0.1% TFA) as eluent A and acetonitrile (0.1% TFA) as eluent B. NMR spectra were recorded on a Bruker Avance III HDTM 400 NMR spectrometer using DMSO-d_6_ or CD_3_OD as solvents and TMS as an internal reference. Mass spectra were obtained by a Bruker Impact 11, UHR-qTOF.

### 4.2. Chemical Synthesis

*(2E,2’E)-N,N’-(Dodecane-1,12-diyl)bis(3-phenylacrylamide)(Bis-cinnamoyl-1,12-dodecamethylenediamine)* (**2**). Trans-cinnamoyl chloride (**1**, 100 mg, 0.6 mmol) was dissolved in dry THF (15 mL), followed by the addition of potassium carbonate (166 mg, 1.2 mmol, 2 equiv). A solution of 1,12-dodecamethylenediamine (60 mg, 0.3 mmol, 0.5 equiv) in THF (10 mL) was added dropwise over 20 min and the reaction mixture was stirred for 4 h. After the completion of the reaction, the crude product was collected by filtration, washed with water, and crystallized from methanol to afford compound **2** as a white solid (125 mg, 45%). The purity was confirmed by analytical HPLC (R_t_ = 26.69 min).

^1^H NMR (400 MHz, DMSO-d_6_): δ (ppm) 1.20–1.31 (br s, 16H, -(CH_2_)_8_), 1.40–1.49 (m, -CH_2_-CH_2_-NH, 4H), 3.12–3.19 (m, -CH_2_NH_2_, 4H), 6.61 (d, *J* = 16 Hz, -CO-CH=CH, 2H), 7.41–7.56 (m, aromatic hydrogens, CH=CHCO, 12H), 8.19 (t, *J* = 5 Hz, 2H, 2NH); ^13^C NMR (100 MHz, DMSO-d_6_): δ (ppm) 165.22 (2 C=O), 138.79 (CH=CHCO), 135.44 (CH=CHCO), 129.82 (aromatic C), 129.39 (aromatic C), 127.92 (aromatic C), 122.85 (aromatic C), 26.90–29.60 (aliphatic CH_2_); HR-MS (ESI-qTOF) (*m*/*z*) (C_30_H_40_N_2_O_2_): calcd 460.3090, found 461.3675 [M + H]^+^, 483.3516 [M + Na]^+^, and 499.3270 [M + K]^+^.

*(2E,2’E)-N,N’-(Dodecane-1,12-diyl)bis(3-(4-hydroxyphenyl)acrylamide* (**4**). To a solution of 4-hydroxycinnamic acid (**3**, 100 mg, 0.6 mmol) in DMF (20 mL) was added *N*-methylmorpholine (NMM, 124 μL, 1.2 mmol, 2 equiv) followed by 1-[bis(dimethylamino)methylene]-1H-1,2,3-triazolo[4,5-b]pyridinium 3-oxide hexafluorophosphate (HATU, 114 mg, 0.3 mmol, 0.5 equiv). The mixture was stirred at room temperature for 20 min, followed by the addition of 1,12-dodecamethylene diamine (60 mg, 0.3 mmol, 0.5 equiv). After 4 h, the solvent was evaporated, and the resulting crude solid was loaded onto silica gel and purified via chromatography using ethyl acetate:methanol as 90:10 (*v*/*v*), to provide compound **4** as a white powder (95 mg, 32%). The purity was confirmed by analytical HPLC (R_t_ = 21.89 min).

^1^H NMR (400 MHz, DMSO-d_6_): δ (ppm) 1.20–1.32 (br s, 16H, -(CH_2_)_8_), 1.37–1.48 (m, 4H), 3.10–3.19 (m, -CH_2_NH_2_, 4H), 6.42 (d, *J* = 15 Hz, -CO-CH=CH, 2H), 6.78–7.37 (m, aromatic hydrogens, CH=CHCO, 10H), 7.95 (t, *J* = 5 Hz, 2H, 2NH), 9.81 (s, 2H, 2OH). ^13^C NMR (100 MHz, DMSO-d*6*): δ (ppm) 165.69 (2 C*=*O), 159.21 (2C*-*OH), 138.89 (Alkene carbons), 129.59 (aromatic C), 126.43 (aromatic C), 119.30 (aromatic C), 116.18 (aromatic C), 26.96–29.68 (aliphatic CH_2_); HR-MS (ESI-qTOF) (*m*/*z*) (C_30_H_40_N_2_O_4_): calcd 492.2988, found 493.3185 [M + H]^+^, 515.3227 [M + Na]^+^, 531.2753 [M + K]^+^.

*(2E,2’E)-N,N’-(Hexane-1,6-diyl)bis(3-(4-hydroxyphenyl)acrylamide)* (**5**). The same synthetic procedure as used in the synthesis of compound **4** is used in the synthesis of compound **5**. 4-Hydroxycinnamic acid (**3**, 100 mg, 0.6 mmol), 1,6-diaminohexane (35 mg, 0.3 mmol, 0.5 equiv), HATU (114 mg, 0.3 mmol, 0.5 equiv), and *N*-methylmorpholine (124 μL, 1.2 mmol, 2 equiv) were used for the synthesis. The crude product was purified by silica gel column chromatography, using ethyl acetate: methanol as 90:10 (*v*/*v*), affording a white product (**5**, 80 mg, 32%).

^1^H NMR (400 MHz, DMSO-d_6_): δ (ppm) 1.26–1.35 (br s, 4H, -(CH_2_)_2_), 1.39–1.49 (m, 4H, -CH_2_-CH_2_-NH), 3.10–3.20 (m, -CH_2_NH_2_, 4H), 6.44 (d, *J* =16 Hz, -CO-CH=CH, 2H), 6.82–7.41 (m, aromatic hydrogens, CH=CHCO, 10H), 7.99 (t, *J* = 6 Hz, 2H, 2NH), 9.84 (s, 2H, 2OH); ^13^C NMR (100 MHz, DMSO-d_6_): 165.70 (2 C*=*O), 159.21 (2 C*-*OH), 138.91 (Alkene carbons), 129.60 (aromatic C), 126.43 (aromatic C), 119.30 (aromatic C), 116.10 (aromatic C), 26.60–29.70 (aliphatic *C*H_2_). HR-MS (ESI-qTOF) (*m*/*z*) (C_24_H_28_N_2_O_4_): calcd 408.2049, found 409.2243 [M + H]^+^, 431.2371 [M + Na]^+^.

*(2E,2’E)-N,N’-(dodecane-1,12-diyl)bis(3-(3-hydroxyphenyl)acrylamide)* (**7**). The synthetic procedure is the same as the procedure used to synthesize compounds **4** and **5**. 3-Hydroxycinnamic acid was used instead of 4-hydroxycinnamic acid. In brief, 3-hydroxycinnamic acid (**6**, 100 mg, 0.6 mmol), 1,12-dodecamethylenediamine (60 mg, 0.3 mmol, 0.5 equiv), HATU (114 mg, 0.3 mmol, 0.5 equiv), *N*-methylmorpholine (124 μL, 1.2 mmol, 2 equiv) were used for the synthesis. The crude product was purified by silica gel column chromatography, using ethyl acetate: methanol as 90:10 (*v*/*v*), affording a white product (**7**, 110 mg, 37%). The purity was confirmed by analytical HPLC (R_t_ = 22.19 min).

^1^H NMR (400 MHz, CD_3_OD): δ (ppm) 1.23–1.36 (br s, 16H, -(CH_2_)_8_), 1.48–1.56 (m, 4H, -CH_2_-CH_2_-NH), 3.20–3.25 (m, -CH_2_NH_2_, 4H), 6.53 (d, *J* =15 Hz, -CO-CH=CH, 2H), 6.80–7.46 (m, aromatic hydrogens, CH=CHCO, 10H); ^13^C NMR (100 MHz, CD_3_OD): δ (ppm) 167.24 (2 C*=*O), 157.61 (2 C*-*OH), 140.30 (CH=CHCO), 136.25 (CH=*C*HCO), 120.38 (aromatic C), 118.89 (aromatic C), 116.47 (aromatic C), 113.70 (aromatic C), 39.20 (CH_2_NH), 26.60–29.25 (alphantic CH_2_); HR-MS (ESI-qTOF) (m/z) (C_30_H_40_N_2_O_4_): calcd 492.2988, found 493.3168 [M + H]^+^, 515.2990 [M + Na]^+^, 531.2733 [M + K]^+^.

*(E)-N-(12-Aminododecyl)-3-(3-hydroxyphenyl) acrylamide* (**8**). 3-Hydroxycinnamic acid (**6**, 200 mg, 1.2 mmol), was added to equimolar amounts of 1,12-dodecamethylenediamine (244 mg, 1.2 mmol) and (2-(1H-benzotriazol-1-yl)-1,1,3,3-tetramethyluronium hexafluorophosphate (HBTU, 455 mg, 1.2 mmol, 1 equiv), in DMF (20 mL), followed by the addition of *N*,*N*-diisopropylethylamine (DIPEA, 417 μL, 2.4 mmol, 2 equiv) and stirring at room temperature for 3 h. The crude compound was subjected to silica gel column chromatography for purification, using ethyl acetate:methanol:ammonia as 73:25:2 (*v*/*v*/*v*), to give a white product (**8**, 115 mg, 27%). The purity was confirmed by analytical HPLC (R_t_ = 19.09 min).

^1^H NMR (400 MHz, DMSO-d_6_): δ (ppm) 1.20–1.35 (br s, 16H, -(CH_2_)_8_), 1.40–1.58 (m, 4H, -CH_2_-CH_2_-NH), 2.68–2.81 (m, 2H, CH_2_NH_2_), 3.10–3.21 (m, 2H, NHCH_2_), 6.55 (d, *J* = 16 Hz, -CO-CH=CH, 1H), 6.76–7.32 (m, aromatic hydrogens, CH=CHCO, 5H), 7.69 (s, 2H, NH_2_), 8.08 (t, *J* = 6 Hz, 1H, NH), 9.57 (s, 1H, OH); ^13^C NMR (100 MHz, DMSO-d_6_): δ (ppm) 164.25 (2 C*=*O), 158.17(C*-*OH), 138.99 (CH=CHCO), 136.69 (CH=CHCO), 130.37 (aromatic C), 122.58 (aromatic C), 119.07 (aromatic C), 117.03 (aromatic C), 114.12 (aromatic C), 22.50–29.65 (-CH_2_). HR-MS (ESI-qTOF) (*m*/*z*) (C_21_H_34_N_2_O_2_): calcd 346.2620, found 347.0936 [M + H]^+^.

*(E)-N-(12-Cinnamamidododecyl)-3-(3-hydroxyphenyl) acrylamide* (**9**). The synthetic procedure is the same as the one used in synthesis of compound **5** using instead *N*-(12-aminododecyl)-3-(3-hydroxyphenyl) acrylamide (**8**, 50 mg, 0.14 mmol), trans-cinnamic acid (**1**, 20 mg, 0.14 mmol), HATU (27 mg, 0.07 mmol, 0.5 equiv), and *N*-methylmorpholine (29 μL, 0.28 mmol, 2 equiv). The crude compound was subjected to silica gel column chromatography to purify the crude product, using methylene chloride:ethylacetate as 70:30 (*v*/*v*) to yield **9** (35 mg, 50%). The purity was confirmed by analytical HPLC (R_t_ = 23.94 min).

^1^H NMR (400 MHz, DMSO-d_6_): δ (ppm) 1.30–1.32 (br s, 16H, -(CH_2_)_8_), 1.38–1.50 (m, 4H, -CH_2_-CH_2_-NH), 3.09–3.20 (m, -CH_2_NH_2_, 4H), 6.50 (d, *J* = 16 Hz, -CO-CH=CH, 1H), 6.59 (d, *J* = 16 Hz, -CO-CH=CH, 1H), 6.76–7.55 (m, aromatic hydrogens, CH=CHCO, 11H), 8.02–8.10 (m, 2H, 2NH), 9.55 (s, 1H, OH). ^13^C NMR (100 MHz, DMSO-d_6_): δ (ppm) 165.23 (C*=*O), 165.22 (C*=*O), 158.14 (C*-*OH), 138.98 (CH=CHCO), 138.79(CH=CHCO), 136.71 (CH=CHCO), 135.45 (CH=CHCO), 130.37 (aromatic C), 129.81 (aromatic C), 129.38 (aromatic C), 127.91 (aromatic C), 122.85 (aromatic C), 122.59 (aromatic C), 119.08 (aromatic C), 117.02 (aromatic C), 114.11 (aromatic C), 26.90–29.62 (aliphatic -CH_2_); HR-MS (ESI-qTOF) (*m*/*z*) (C_30_H_40_N_2_O_3_): calcd 476.3039, found 477.3245 [M + H]^+^, 499.3068 [M + Na]^+^.

*N-(12-Aminododecyl)cinnamamide* (**10**). HBTU (507 mg, 1.34 mmol, 1 equiv) was added to equimolar amounts of trans-cinnamic acid (**1**, 200 mg, 1.34 mmol) and 1,12-dodecamethylenediamine (268 mg, 1.34 mmol, 1 equiv) in DMF (20 mL), followed by the addition of DIPEA (465 μL, 2.4 mmol, 2 equiv) and stirring at room temperature for 3 h. After completion of the reaction, the solution was evaporated. The white residue powder was subjected to silica gel column chromatography, using ethyl acetate:methanol:ammonia as 75:23:2 (*v*/*v*/*v*) to give the desired product (**10**) (110 mg, 25%). The purity was confirmed by analytical HPLC (R_t_ = 20.28 min).

^1^H NMR (400 MHz, DMSO-d_6_): δ (ppm) 1.21–1.32 (br s, 16H, -(CH_2_)_8_), 1.39–1.55 (m, 4H, -CH_2_-CH_2_-NH), 2.74 (t, *J* = 8 Hz, 2H, CH_2_NH_2_), 3.18 (q, *J* = 7 Hz, 2H, NHCH_2_), 6.60 (d, *J* = 16 Hz, -CO-CH=CH, 1H), 7.35–7.55 (m, aromatic hydrogens, CH=CHCO, 6H), 7.68 (s, 2H, NH_2_), 8.09 (t, *J* = 6 Hz, 1H, NH); ^13^C NMR (100 MHz, DMSO-d_6_): δ (ppm) 165.23 (CO), 138.79 (CH=CHCO), 135.43 (CH=CHCO), 129.83 (aromatic C), 129.39 (aromatic C), 127.91 (aromatic C), 122.88 (aromatic C), 26.20–29.70 (aliphatic -CH_2_); HR-MS (ESI-qTOF) (*m*/*z*) (C_21_H_34_N_2_O): calcd 330.2671, found 331.3007 [M + H]^+^.

*(E)-N-(12-Cinnamamidododecyl)-3-(4-hydroxyphenyl) acrylamide* (**11**). The synthetic procedure was the same as the one used for the synthesis of **5** using *N*-(12-aminododecyl) cinnamamide (**10**, 50 mg, 0.15 mmol), 4-hydroxycinnamic acid (3, 25 mg, 0.15 mmol, 1 equiv), HATU (29 mg, 0.07 mmol, 0.5 equiv), and *N*-methylmorpholine (23 μL, 0.28 mmol, 2 equiv). Silica gel column chromatography was used for the purification of the crude product with methylene chloride:ethyl acetate (70:30) (*v*/*v*) as eluents to generate compound **11** as a white product (40 mg, 55%). The purity was confirmed by analytical HPLC (R_t_ = 23.68 min).

^1^H NMR (400 MHz, DMSO-d_6_): δ (ppm) 1.15–1.33 (br s, 16H, -(CH_2_)_8_), 1.32–1.50 (m, 4H, -CH_2_-CH_2_-NH), 3.07–3.21 (m, -CH_2_NH_2_, 4H), 6.41 (d, *J* = 16 Hz, -CO-CH=CH, 1H), 6.59 (d, *J* = 16 Hz, -CO-CH=CH, 1H), 6.77–7.55 (m, aromatic hydrogens, CH=CHCO, 11H), 7.92 (t, *J* = 5 Hz, 1H, NH), 8.08 (t, *J* = 5 Hz, 1H, NH), 9.80 (s, 1H, OH); ^13^C NMR (100 MHz, DMSO-d_6_): δ (ppm) 164.95 (C*=*O), 164.20 (C*=*O), 159.21 (C-OH), 138.88 (CH=CHCO), 138.79 (CH=CHCO), 135.45 (CH=CHCO), 129.80 (aromatic C), 129.58 (aromatic C), 127.91 (aromatic C), 126.43 (aromatic C), 122.86 (aromatic C), 119.31 (aromatic C), 116.18 (aromatic C), 23.50–29.70 (aliphatic -CH_2_); HR-MS (ESI-qTOF) (*m*/*z*) (C_30_H_40_N_2_O_3_): calcd 476.3039, found 477.3203 [M + H]^+^, 499.3364 [M + Na]^+^.

*(2E,2’E)-N,N’-(hexane-1,6-diyl)bis(3-phenylacrylamide)* (**12**). Hexane-1,6-diamine (0.58 g, 5 mmol), cinnamic acid (1.48 g, 10 mmol), and pyridine (15 mL) were mixed in a round bottom flask and stirred for 15–20 min. Triphenyl phosphite (2.8 mL, 10.75 mmol) was added to the flask, and the reaction mixture was refluxed for 8 h. Pyridine was distilled out after cooling the mixture to room temperature to reduce the volume to 5 mL. The mixture was left for overnight standing. After acetone was added, the obtained solid product was filtered and washed with acetone. Methanol was used for the recrystallization of the crude product (1.32 g, 70%). M.P. 192 °C.

^1^H NMR (400 MHz, DMSO-*d_6_*): δ 1.33 (s, 4H, CH_2_); 1.47 (s, 4H, -CH_2_-CH_2_-NH), 4.15 (q, 4H, *J* = 19.4 Hz, -CH_2_NH_2_), 6.63 (d, *J* = 15.8 Hz, 2H, Olefin proton), 7.35–7.43 (m, 4H, Olefin protons and Ph-H); 7.52 (m, 8H, PhH); 8.10 (s, 2H, NH). ^13^C NMR (400 MHz, DMSO-*d_6_*) δ 165.30 (2 C*=*O), 138.81 (CH=CHCO), 135.42 (CH=CHCO), 129.88 (aromatic C), 129.39 (aromatic C), 127.92 (aromatic C), 122.85 (aromatic C), 39.09 (CH_2_NH), 29.6 (-CH_2_), 26.7 (-CH_2_), HR-MS (ESI-qTOF) (m/z) (C_24_H_29_N_2_O_2_): calcd 376.2151, found 377.2240 [M + H]^+^.

### 4.3. Three-Dimensional Virtual Docking Using Molsoft ICM-Pro System

The in silico molecular docking was performed using Molsoft ICM-Pro (x64) [33]. The druggable binding cavity located in the redox-regulatory domain of the APE/Ref-1 protein (1BIX, 10.2210/pdb1BIX/pdb) was identified and used for docking. The molecular docking conformation presenting the lowest binding energy was selected to visualize the possible compound-protein interaction. The virtual docking scores were collected and the ligands with lower observed ICM scores were chosen due to the higher chance of the ligand being a binder to the APE/Ref-1 cavity [50].

### 4.4. Cell Lines and Cell Culture

Human malignant melanoma cells (A375, #CRL-1619; SK-Mel-28, #HTB-72) were purchased from ATCC. Cells were cultured in Dulbecco’s Modified Eagle Medium (DMEM, ATCC #30-2002; for A375 cells) or Eagles’ Minimum Essential Medium (EMEM, ATCC #30-2003; for SK-Mel-28 cells) supplemented with 10% fetal bovine serum (FBS, ATCC #30-2020) at 37 °C in 5% CO_2_. All cell lines were passaged once reaching 70–90% confluence.

Human immortalized melanoblast cell lines Hermes 1, Hermes 3A, and Hermes 4A were obtained from the Wellcome Trust Functional Genomics Cell Bank, generously supplied to researchers [38]. Cells were cultured in the medium as recommended containing RPMI 1640, FBS (10%), 12-O-tetradecanoyl phorbol acetate (TPA, 200 nM), Cholera toxin (CT, 200 pM), human stem cell factor (hSCF, 10 ng/mL), and endothelin 1 (EDN1, 10 nM) [51].

### 4.5. In Vitro Anti-Melanoma Activity Screening Using MTT Colorimetric Assay

Cell viability analysis was conducted based on the bio-reduction of a tetrazolium compound (3-(4,5-dimethylthiazol-2-yl)-5-(3-carboxymethoxyphenyl)-2-(4-sulfophenyl)-2H-tetrazolium) (MTT) metabolized by live cells. Briefly, human melanoma cells were inoculated into 96 well microtiter plates in 100 μL at plating densities ranging from 5000 to 8000 cells/well depending on the doubling time of individual cell lines (A375: 5000 cells/well; Sk-Mel-28: 7500 cells/well; Hermes: 8000 cells/well). After cell inoculation, the microtiter plates are incubated for 24 h prior to the addition of experimental drugs. Various drugs dissolved in DMSO were added to a serum-free medium at different concentrations and incubated for 72 h, and negative controls were treated with the equivalent amounts of the DMSO solution. By the end of treatment, MTT solution was added to the wells to a final concentration of 0.5 mg/mL for an additional 2 h incubation. Solubilization solution (4 mM HCl, 0.1% NP-40 in isopropanol) was then added, and the absorbance was recorded at 595 nm on a plate reader (BioRad, Hercules, CA, USA). All readings were compared to the control, which represented 100% viability. Each experiment was performed in triplicate and independently repeated at least two times.

### 4.6. Luciferase Assay to Determine AP-1 Transactivation Activities

3 × AP1pGL3 (3 × AP-1 in pGL3-basic) was a gift from Alexander Dent (Addgene plasmid #40342), which contains three canonical AP-1 binding sites (TGACTCA) upstream of a minimal promoter fragment containing a TATA box in the luciferase reporter plasmid pGL3-basic [39]. 3 × AP1pGL3 was transfected to human melanoma A375 cells using Lipofectamine 2000 following the manufacturer’s instructions (Invitrogen). 24 h after transfection, the cells were treated with H_2_O_2_ (100 µM) for 48 h in the presence or absence of APE/Ref-1 inhibitors (10 µM).

After different treatments, the cells were rinsed twice with phosphate-buffered saline (PBS). Cells were then scraped from the plates in PBS and pelleted for 4 min at 4 °C in a microcentrifuge at 12,000 rpm. Cell pellets were resuspended in 100 µL luciferase lysis buffer as per manufacturer instructions (Promega, #E1500). Luciferase activity measured in relative light units reflects the AP-1 transcription rate and was detected by a SpectraMax M5 UV VIZ Plate Reader. A cell count was conducted for normalization. All samples were studied in duplicate, and readings were taken in triplicate. Each experiment was repeated at least two times.

### 4.7. In Vivo Xenograft Melanoma

The Institutional Animal Care and Use Committee approved all the animal procedures at Chapman University (IACUC #2020-1131). Male nude mice (Nu/Nu) were purchased from Charles River (Wilmington, MA, USA) and were housed and maintained in the Chapman University vivarium under pathogen-free conditions. Human metastatic melanoma A375 cells were injected subcutaneously into the flank (1 × 10^6^ cells per mouse). Three days-post tumor cell injection, mice were randomized into different groups. The treatment group was injected with compound **2** (5 mg/kg/day, IP) for 21 days. The growth of the tumors was monitored three times a week and measured using digital Vernier calipers. The size of the tumors was calculated as tumor volume (mm^3^) = (L × W^2^)/2. The mice were sacrificed at the end of the study via CO_2_ euthanasia and cervical dislocation.

## 5. Conclusions

The novel APE/Ref-1 inhibitor developed in our study (compound **2**) showed promising anti-melanoma activities in vitro and in vivo, suggesting that targeting APE/Ref-1-mediated signaling might be a novel and effective strategy for melanoma therapy. More studies are warranted in the future to develop more bioavailable and potent APE/Ref-1 inhibitors with chemical modifications of the lead compound.

## Figures and Tables

**Table 1:** Docking scores and cytotoxicity of compounds in human melanoma A375 and SK-Mel-28 cells.

Synthesized Compounds	Docking Score	Anti-Melanoma Activities
2	−9.23	Cytotoxicity is not dose-dependent; at 0.1 µM, cell viability reduced to <50% of control in A375 cells.
11	−16.31	0.079 ± 0.013 µM (IC_50_, detected by MTT assay, average in two human melanoma cell lines: A375 and SK-Mel-28)
4	−20.14	50% inhibition of cell survival not observed up to 10 µM.
5	−12.91	50% inhibition of cell survival not observed up to 50 µM.
7	−11.74	50% inhibition of cell survival not observed up to 10 µM.
8	−12.51	50% inhibition of cell survival not observed up to 25 µM.
9	−5.97	50% inhibition of cell survival not observed up to 10 µM.
10	−15.44	50% inhibition of cell survival not observed up to 25 µM.
12	−10.06	50% inhibition of cell survival not observed up to 10 µM.

## Data Availability

Not applicable.

## Supplementary Materials

The following supporting information can be downloaded at: https://www.mdpi.com/article/10.3390/molecules27092672/s1, Analytical HPLC profile of representative compounds; Figure S1: ^1^H NMR (400 MHz, DMSO-d_6_) of (2E,2’E)-bis-cinnamoyl-1,12-dodecamethylenediamine (**2**); Figure S2: ^13^C NMR (100 MHz, DMSO-d_6_) of (2E,2’E)-bis-cinnamoyl-1,12-dodecamethylenediamine (**2**); Figure S3: Mass spectra of (2E,2’E)-bis-cinnamoyl-1,12-dodecamethylenediamine (**2**); Figure S4: ^1^H NMR (400 MHz, DMSO-d6) of (2E,2’E)-*N*,*N’*-(dodecane-1,12-diyl)bis(3-(4 hydroxyphenyl)acrylamide (**4**); Figure S5: ^13^C NMR (100 MHz, DMSO-d_6_) of (2E,2’E)-*N*,*N’*-(dodecane-1,12-diyl)bis(3-(4-hydroxyphenyl) acrylamide (**4**); Figure S6: Mass spectra of (2E,2’E)-*N*,*N’*-(dodecane-1,12-diyl)bis(3-(4-hydroxyphenyl) acrylamide (**4**); Figure S7: ^1^H NMR (400 MHz, DMSO-d_6_) of (2E,2’E)-*N*,*N’*-(hexane-1,6-diyl)bis(3-(4-hydroxyphenyl)acrylamide) (**5**); Figure S8: ^13^C NMR (100 MHz, DMSO-d_6_) of (2E,2’E)-*N*,*N’*-(hexane-1,6-diyl) bis(3-(4-hydroxyphenyl) acrylamide) (**5**); Figure S9: Mass spectra of (2E,2’E)-*N*,*N’*-(hexane-1,6-diyl) bis(3-(4-hydroxyphenyl) acrylamide) (**5**); Figure S10: ^1^H NMR (400 MHz, DMSO-d_6_) of (2E,2’E)-*N*,*N’*-(dodecane-1,12-diyl)bis(3-(3 hydroxyphenyl)acrylamide) (**7**); Figure S11: ^13^C NMR (100 MHz, DMSO-d_6_) of (2E,2’E)-*N*,*N’*-(dodecane-1,12-diyl) bis(3-(3-hydroxyphenyl) acrylamide) (**7**); Figure S12: Mass spectra of (2E,2’E)-*N*,*N’*-(dodecane-1,12-diyl) bis(3-(3-hydroxyphenyl) acrylamide) (**7**); Figure S13: ^1^H NMR (400 MHz, DMSO-d_6_) of (E)-*N*-(12-aminododecyl)-3-(3-hydroxyphenyl) (**8**); Figure S14: ^13^C NMR (100 MHz, DMSO-d_6_) of (E)-*N*-(12-aminododecyl)-3-(3-hydroxyphenyl) (**8**); Figure S15: Mass spectra of (E)-*N*-(12-aminododecyl)-3-(3-hydroxyphenyl) (**8**); Figure S16: ^1^H NMR (400 MHz, DMSO-d_6_) of (E)-*N*-(12-cinnamamidododecyl)-3-(3-hydroxyphenyl) acrylamide (**9**); Figure S17: ^13^C NMR (100 MHz, DMSO-d_6_) of (E)-*N*-(12-cinnamamidododecyl)-3-(3-hydroxyphenyl) acrylamide (**9**); Figure S18: Mass spectra of (E)-*N*-(12-cinnamamidododecyl)-3-(3-hydroxyphenyl) acrylamide (**9**); Figure S19: ^1^H NMR (400 MHz, DMSO-d6) of N-(12-aminododecyl) cinnamamide (**10**); Figure S20: ^13^C NMR (100 MHz, DMSO-d_6_) of *N*-(12-aminododecyl) cinnamamide (**10**); Figure S21: Mass spectra of *N*-(12-aminododecyl) cinnamamide (10); Figure S22: ^1^H NMR (400 MHz, DMSO-d_6_) of (E)-*N*-(12-cinnamamidododecyl)-3-(4-hydroxyphenyl) (**11**); Figure S23: ^13^C NMR (100 MHz, DMSO-d6) of (E)-*N*-(12-cinnamamidododecyl)-3-(4-hydroxyphenyl) (**11**); Figure S24: Mass spectra of (E)-*N*-(12-cinnamamidododecyl)-3-(4-hydroxyphenyl) (**11**); Figure S25: ^1^H NMR (400 MHz, DMSO-d_6_) of (2E,2’E)-*N*,*N’*-(hexane-1,6-diyl)bis(3-phenylacrylamide) (**12**); Figure S26: 13C NMR (100 MHz, DMSO-d6) of (2E,2’E)-N,N’-(hexane-1,6-diyl)bis(3-phenylacrylamide) (**12**); Figure S27: No significant reduction of body weight was evident in mice treated with compound **2** (5 mg/kg*,* IP *,* daily) for 21 days in a melanoma xenograft mouse model.

## Sample Availability

Samples of the compounds are available from the authors.
