# Supplementary material for: Bis-Cinnamamide Derivatives as APE/Ref-1 Inhibitors for the Treatment of Human Melanoma
Source: Molecules. 2022 Apr 21;27(9):2672. doi: 10.3390/molecules27092672 (PMC9103902; doi:10.3390/molecules27092672)
Supplement: Supplementary file 1 [file molecules-27-02672-s001.zip › molecules-1650919-supplementary.pdf]

Supplementary Materials

Bis-Cinnamamide Derivatives as APE/Ref-1 Inhibitors for the Treatment of Human Melanoma

Razan Alhazmi [1,#], Shirley Tong [1,#], Shaban Darwish [1,§], Elina Khanjani [1], Bharti Khungar [1, ξ], Swati Chawla [1], Zhonghui Zheng [2], Richard Chamberlin [2], Keykavous Parang [1,*], Sun Yang [3,*]

[1] *Center for Targeted Drug Delivery, Department of Biomedical and Pharmaceutical Sciences, Chapman University School of Pharmacy, Irvine, California, USA*

[2] *Department of Pharmaceutical Sciences, University of California Irvine, Irvine, California, USA*

[3] *Department of Pharmacy Practice, Chapman University School of Pharmacy, Irvine, California, USA*

[§] *Current address: Organometallic and Organometalloid Chemistry Department, National Research Centre, El Bohouth St, Dokki, Giza, Egypt.*

ξ *Current address: Department of Chemistry, Birla Institute of Technology & Science, Pilani, India*

[#] *Co-First author with equal contribution to the study.*

*Corresponding Authors

Sun Yang, B.Pharm, Ph.D., R.Ph, BCPPS, APH
Chapman University School of Pharmacy
Harry and Diane Rinker Health Science Campus
#297Y, 9401 Jeronimo Road
Irvine, CA 92618, USA
Tel: (714) 516-5418. Fax: (714) 516-5481
E-mail: syang@chapman.edu

Keykavous Parang, Pharm.D., Ph.D.
Chapman University School of Pharmacy
Harry and Diane Rinker Health Science Campus
#262, 9401 Jeronimo Road
Irvine, CA 92618, USA
Tel: (714) 516-5489. Fax: (714) 516-5481
E-mail: parang@chapman.edu

Table of Contents | **Page**

Table of Contents	Page
Analytical HPLC profile of representative compounds	3
^{1}H NMR, ^{13}C NMR, and Mass Spectra of Compounds	10
Effects of compound **2** on mouse body weight after 21 days of treatment	28

Analytical HPLC profile of representative compounds

The analytical HPC was conducted on Shimadzu RP-HPLC system and C18 column (250 cm × 4.60 mm) using water (0.1% TFA) as eluent A and acetonitrile (0.1 % TFA) as eluent B, over 80 min.

(2E,2´E)-N,N'-(Dodecane-1,12-diyl)bis(3-phenylacrylamide)(Bis-cinnamoyl-1,12-dodecamethylenediamine) (2)

12/22/2017 11:03:26 AM Page 1 / 1

SHIMADZU LabSolutions **Analysis Report**

<Sample Information>

Sample Name	: 460		
Sample ID	: 460		
Data Filename	: 460_460_01.lcd		
Method Filename	: 330-346.lcm		
Batch Filename	: 460-493-pOH.lcb		
Vial #	: 1-1	Sample Type	: Unknown
Injection Volume	: 50 uL		
Date Acquired	: 12/22/2017 9:27:15 AM	Acquired by	: darwish
Date Processed	: 12/22/2017 10:47:16 AM	Processed by	: darwish

<Chromatogram>

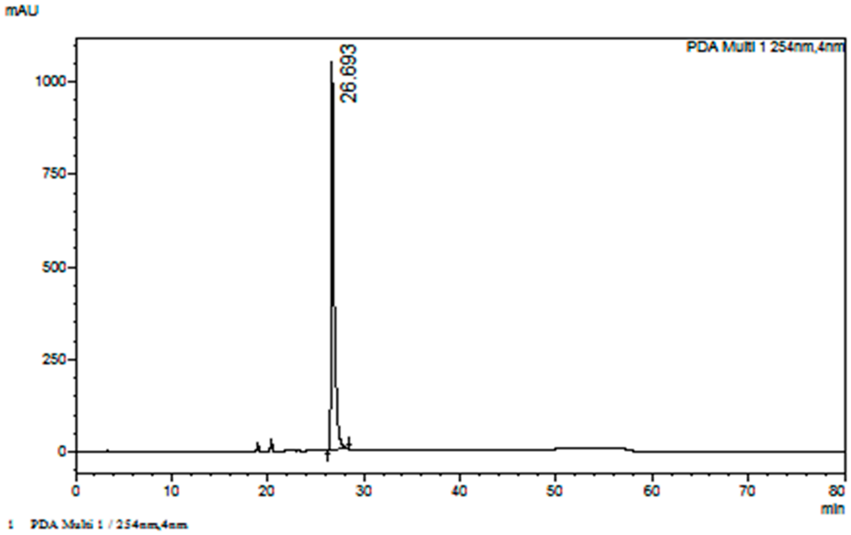

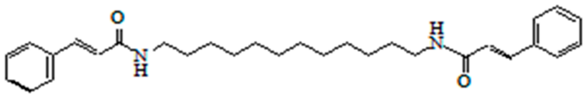

C:\LabSolutions\Data\SH 11917\CINN-DIAMINE\460_460_01.lcd

(2E,2´E)-N,N'-(Dodecane-1,12-diyl)bis(3-(4-hydroxyphenyl)acrylamide (4)

12/22/2017 4:09:00 PM Page 1 / 1

SHIMADZU LabSolutions

Analysis Report

<Sample Information>

Sample Name	: 493-pOH		
Sample ID	: 493-pOH		
Data Filename	: 493-pOH_493-pOH_02.lcd		
Method Filename	: 330-346.lcm		
Batch Filename	: 460-493-pOH.lcb		
Vial #	: 1-2	Sample Type	: Unknown
Injection Volume	: 50 uL		
Date Acquired	: 12/22/2017 10:47:38 AM	Acquired by	: darwish
Date Processed	: 12/22/2017 12:07:40 PM	Processed by	: darwish

<Chromatogram>

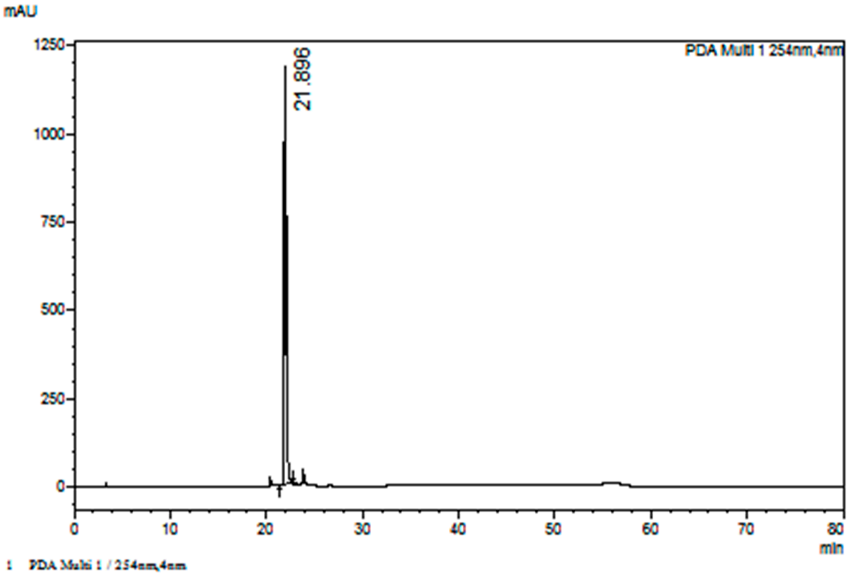

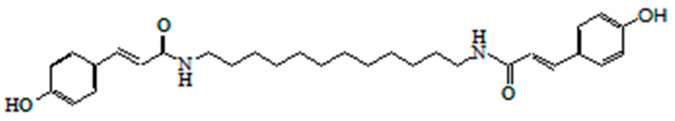

C:\LabSolutions\Data\SH 11917\CINN-DIAMINE\493-pOH_493-pOH_02.lcd

(2E,2'E)-N,N'-(dodecane-1,12-diyl)bis(3-(3-hydroxyphenyl)acrylamide) (7)

12/21/2017 5:59:37 PM Page 1 / 1

SHIMADZU LabSolutions

Analysis Report

<Sample Information>

Sample Name	: 493-mOH		
Sample ID	: 493-mOH		
Data Filename	: 493-mOH1.lcd		
Method Filename	: 330-346.lcm		
Batch Filename	:		
Vial #	: 1-1	Sample Type	: Unknown
Injection Volume	: 50 uL		
Date Acquired	: 12/21/2017 4:29:56 PM	Acquired by	: darwish
Date Processed	: 12/21/2017 5:49:57 PM	Processed by	: darwish

<Chromatogram>

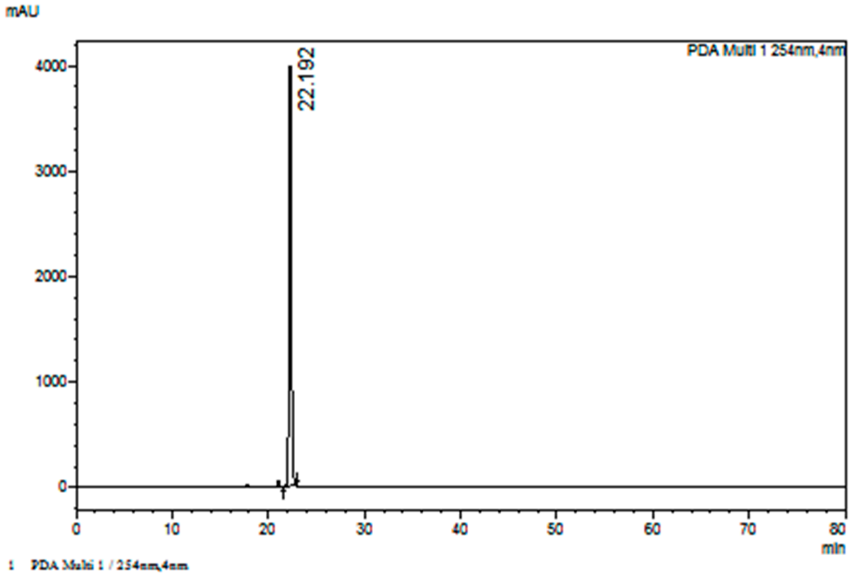

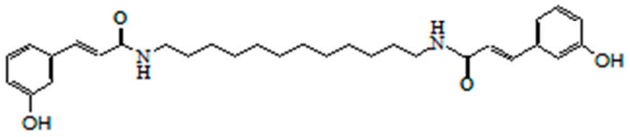

C:\LabSolutions\Data\SH 11917\CINN-DIAMINE\493-mOH1.lcd

(E)-N-(12-Aminododecyl)-3-(3-hydroxyphenyl) acrylamide (8)

SHIMADZU LabSolutions

Analysis Report

<Sample Information>

Sample Name	: 346-mOH		
Sample ID	: 346-mOH		
Data Filename	: 346-mOH1.lcd		
Method Filename	: 330-346.lcm		
Batch Filename	:		
Vial #	: 1-1	Sample Type	: Unknown
Injection Volume	: 50 uL		
Date Acquired	: 12/21/2017 5:57:51 PM	Acquired by	: darwish
Date Processed	: 12/21/2017 7:17:52 PM	Processed by	: darwish

<Chromatogram>

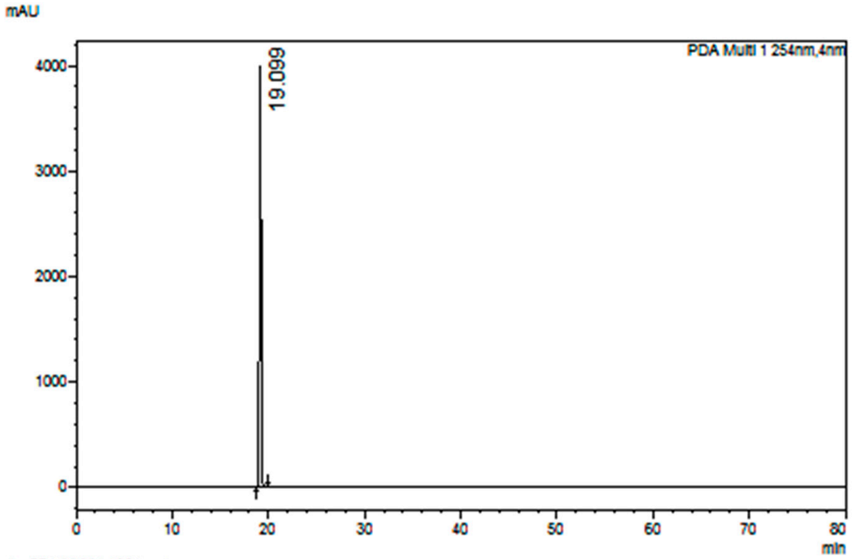

1 PDA Multi 1 / 254nm,4nm

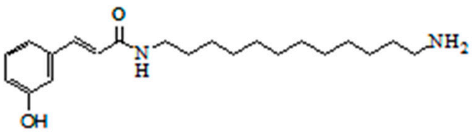

C:\LabSolutions\Data\SH 11917\CINN-DIAMINE\346-mOH1.lcd

(E)-N-(12-Cinnamamidododecyl)-3-(3-hydroxyphenyl) acrylamide (9)

12/21/2017 10:40:52 AM Page 1 / 1

SHIMADZU LabSolutions

Analysis Report

<Sample Information>

Sample Name	: 476-mOH		
Sample ID	: 476-mOH		
Data Filename	: 476-mOH1.lcd		
Method Filename	: 330-346.lcm		
Batch Filename	:		
Vial #	: 1-1	Sample Type	: Unknown
Injection Volume	: 50 uL		
Date Acquired	: 12/21/2017 7:47:53 AM	Acquired by	: darwish
Date Processed	: 12/21/2017 9:25:57 AM	Processed by	: darwish

<Chromatogram>

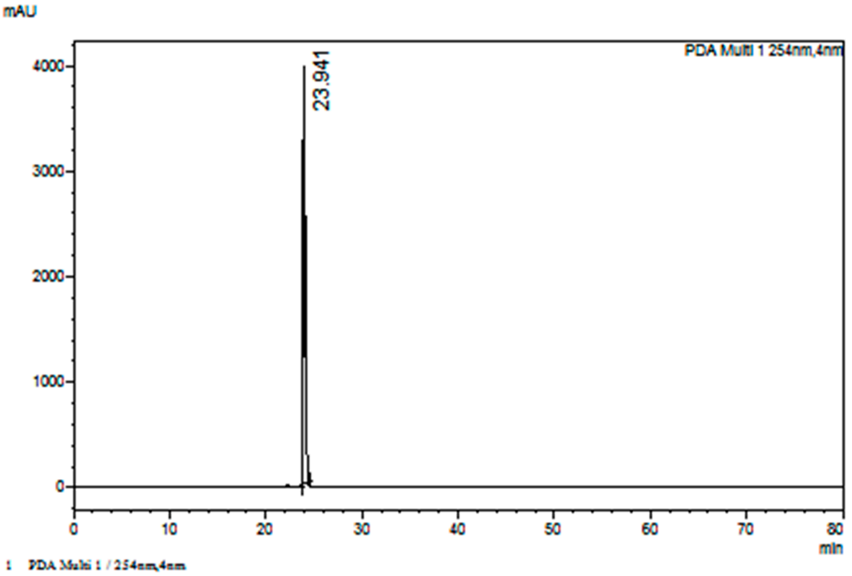

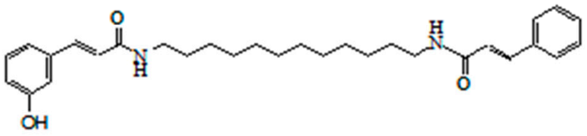

C:\LabSolutions\Data\SH 11917\CINN-DIAMINE\476-mOH1.lcd

N-(12-Aminododecyl)cinnamamide (10)

12/21/2017 10:39:45 AM Page 1 / 1

SHIMADZU LabSolutions

Analysis Report

<Sample Information>

Sample Name	: 330-2		
Sample ID	: 330-2		
Data Filename	: 330-3.lcd		
Method Filename	: 330-346.lcm		
Batch Filename	:		
Vial #	: 1-1	Sample Type	: Unknown
Injection Volume	: 50 uL		
Date Acquired	: 12/21/2017 9:18:19 AM	Acquired by	: darwish
Date Processed	: 12/21/2017 10:38:21 AM	Processed by	: darwish

<Chromatogram>

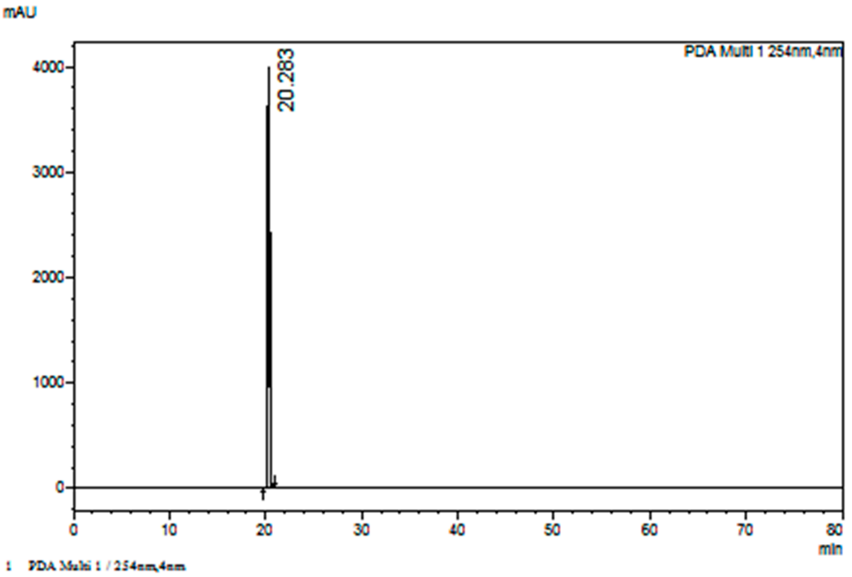

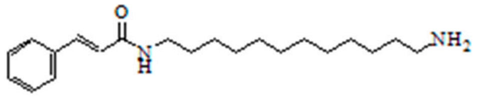

C:\LabSolutions\Data\SH 11917\CINN-DIAMINE\330-3.lcd

(E)-N-(12-Cinnamamidododecyl)-3-(4-hydroxyphenyl) acrylamide (11)

1/27/2018 2:54:23 PM Page 1 / 1

SHIMADZU LabSolutions

Analysis Report

<Sample Information>

Sample Name	: 476-pOH3		
Sample ID	: 476-pOH3		
Data Filename	: 476-pOH4.lcd		
Method Filename	: 330-346.lcm		
Batch Filename	:		
Vial #	: 3-1	Sample Type	: Unknown
Injection Volume	: 10 uL		
Date Acquired	: 1/27/2018 1:13:53 PM	Acquired by	: darwish
Date Processed	: 1/27/2018 2:33:55 PM	Processed by	: darwish

<Chromatogram>

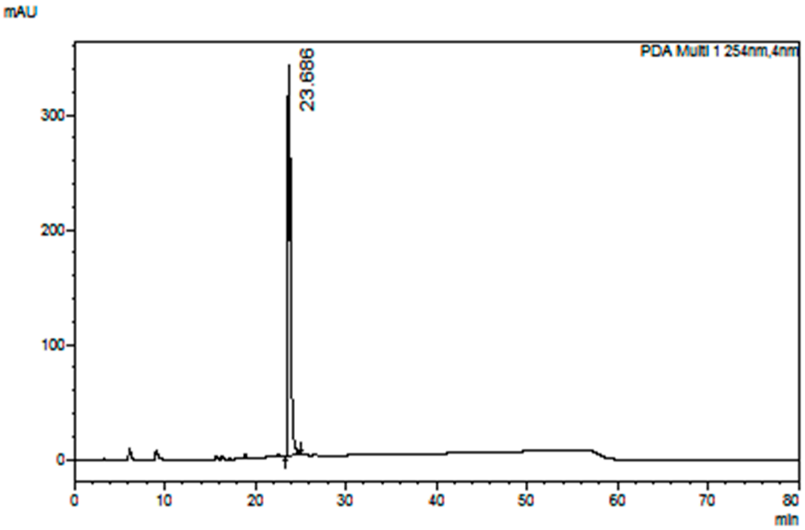

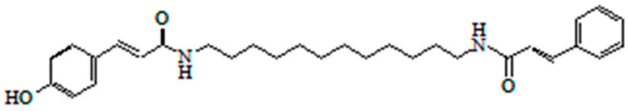

C:\LabSolutions\Data\SH11917\CINN-DIAMINE\476-pOH4.lcd

^{1}H NMR, ^{13}C NMR, and Mass Spectra of Compounds

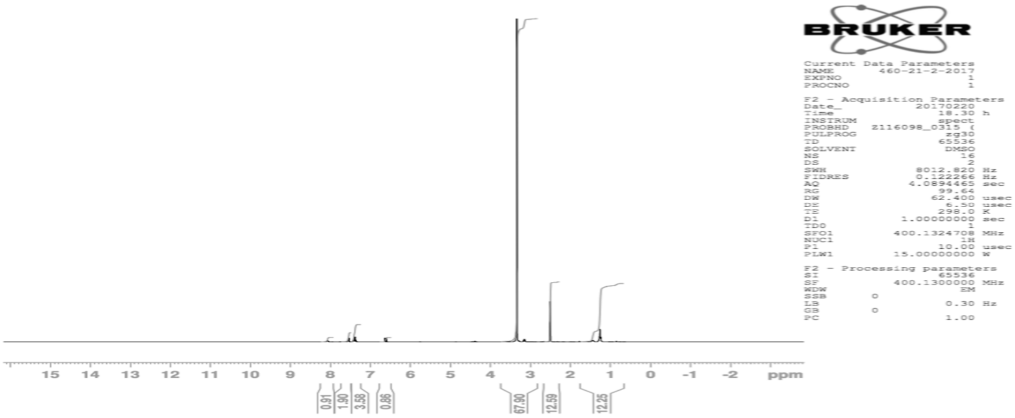

Figure S1. ^{1}H NMR (400 MHz, DMSO-d$_6$) of (2E,2´E)-bis-cinnamoyl-1,12-dodecamethylenediamine (**2**).

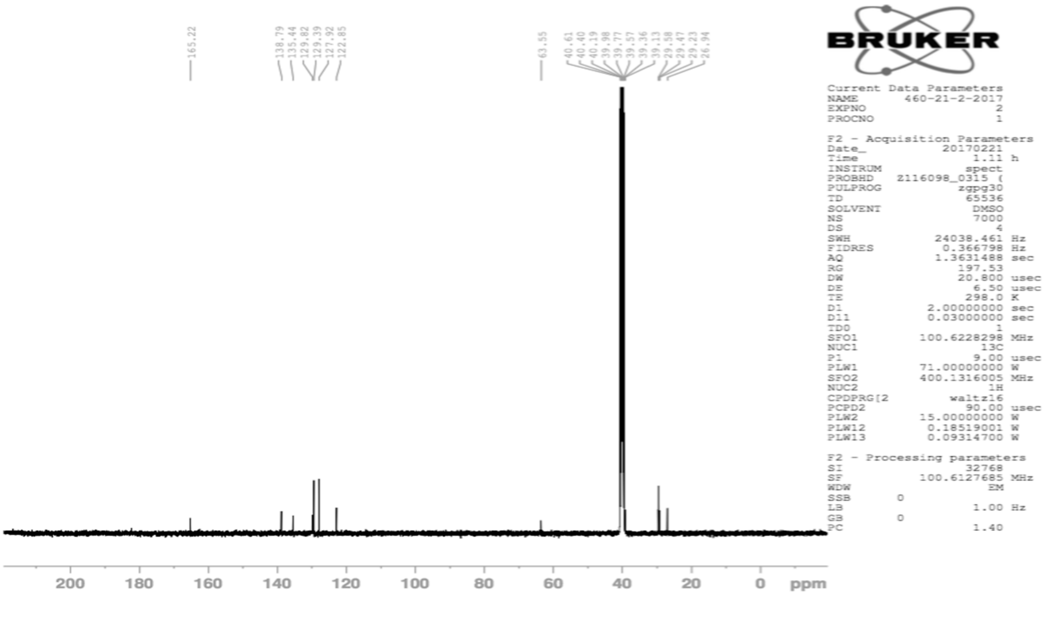

Figure S2. ^{13}C NMR (100 MHz, DMSO-d$_6$) of (2E,2´E)-bis-cinnamoyl-1,12-dodecamethylenediamine (**2**).

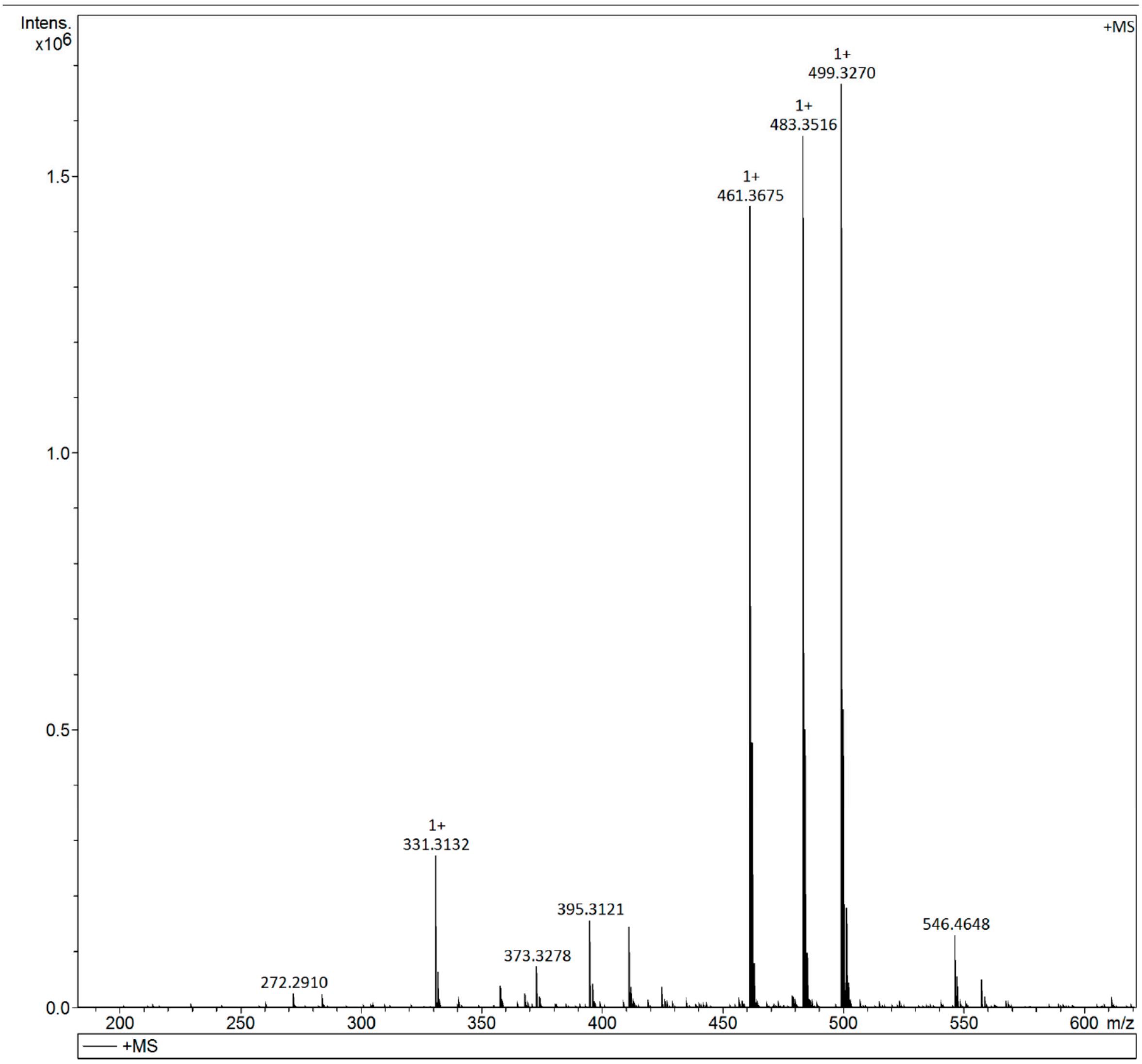

Figure S3. Mass spectra of (2E,2´E)-bis-cinnamoyl-1,12-dodecamethylenediamine (**2**).

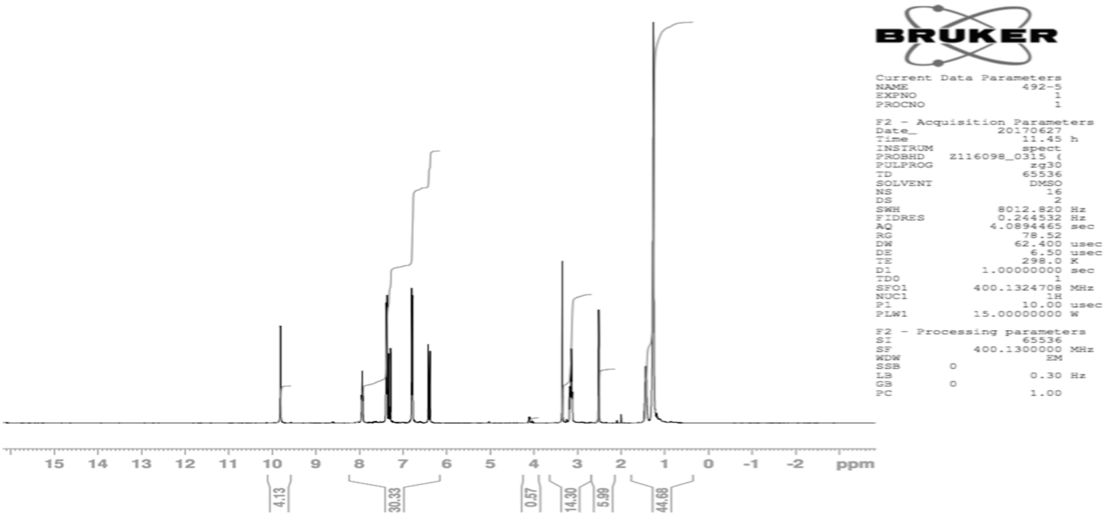

Figure S4. ^{1}H NMR (400 MHz, DMSO-d$_6$) of (2E,2´E)-*N*,*N′*-(dodecane-1,12-diyl)bis(3-(4 hydroxyphenyl)acrylamide (**4**).

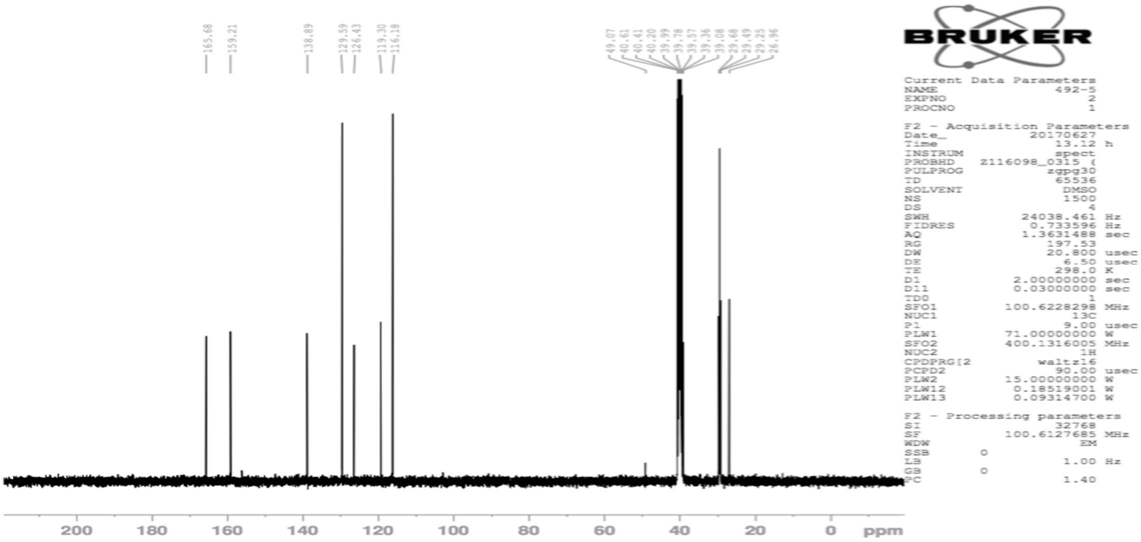

Figure S5. ^{13}C NMR (100 MHz, DMSO-d$_6$) of (2E,2´E)-*N*,*N′*-(dodecane-1,12-diyl)bis(3-(4-hydroxyphenyl) acrylamide (**4**).

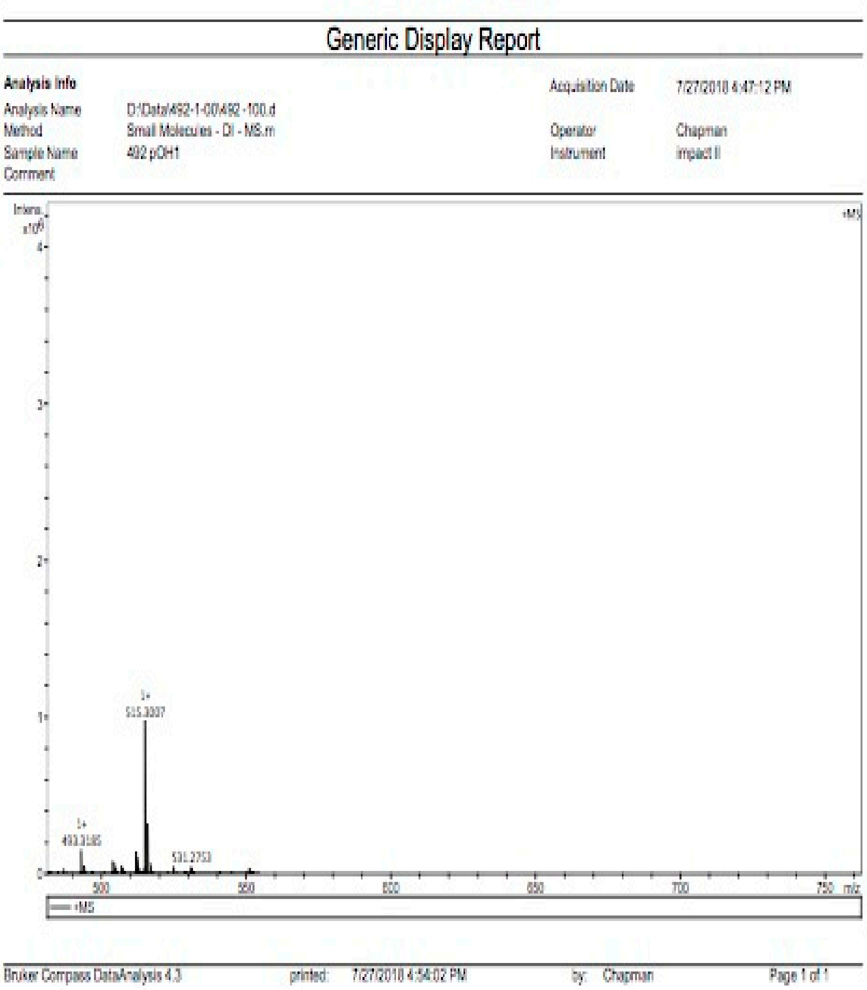

Figure S6. Mass spectra of (2E,2´E)-*N*,*N′*-(dodecane-1,12-diyl)bis(3-(4-hydroxyphenyl) acrylamide (**4**).

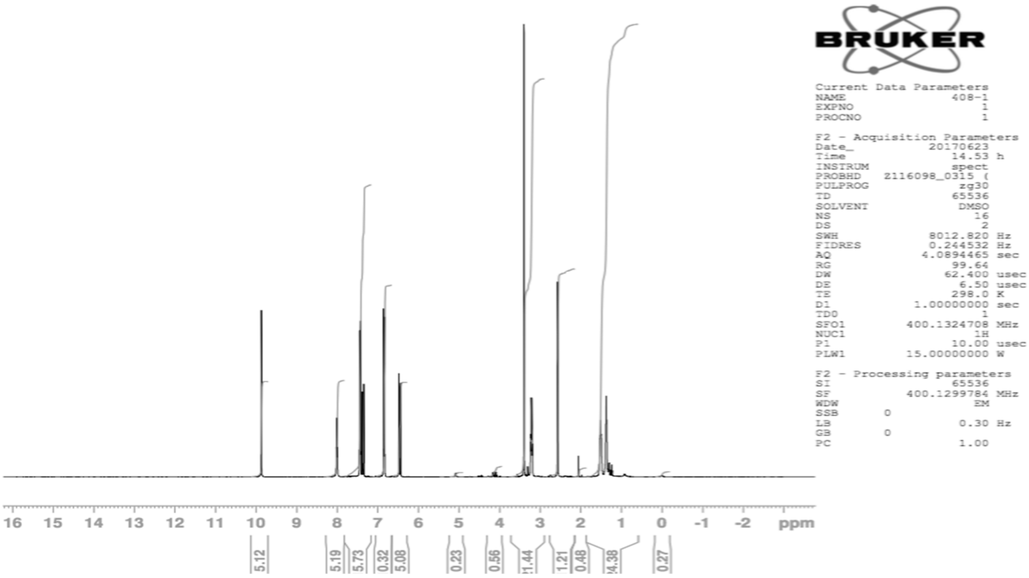

Figure S7. ^{1}H NMR (400 MHz, DMSO-d$_6$) of (2E,2´E)-*N*,*N′*-(hexane-1,6-diyl)bis(3-(4-hydroxyphenyl)acrylamide) (**5**).

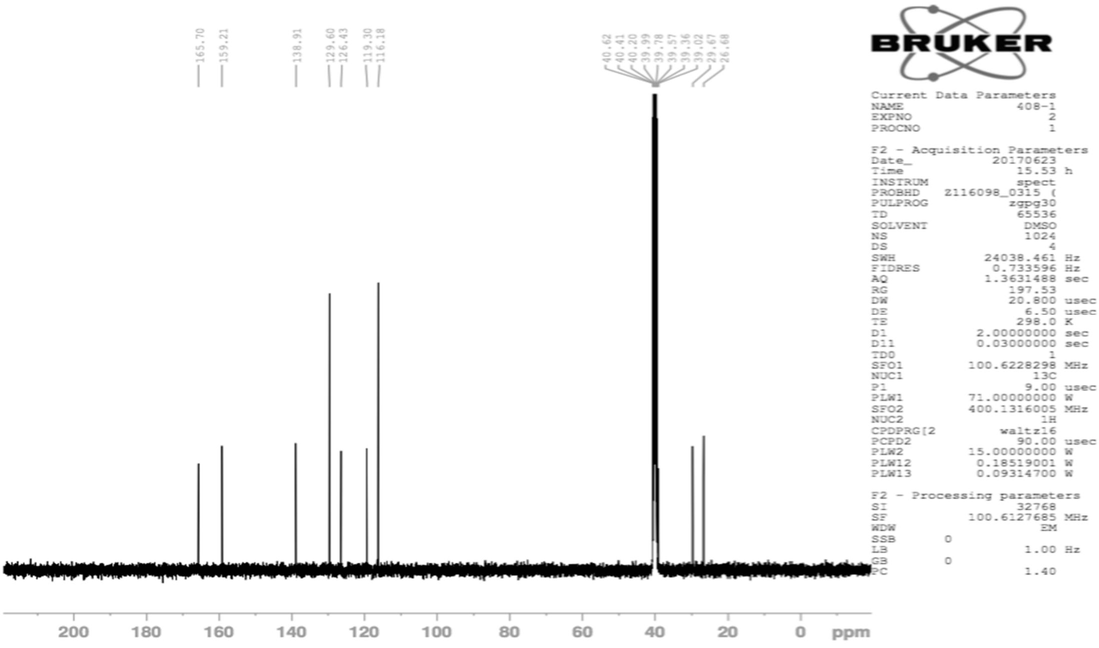

Figure S8. ^{13}C NMR (100 MHz, DMSO-d$_6$) of (2E,2´E)-*N*,*N′*-(hexane-1,6-diyl) bis(3-(4-hydroxyphenyl) acrylamide) (**5**).

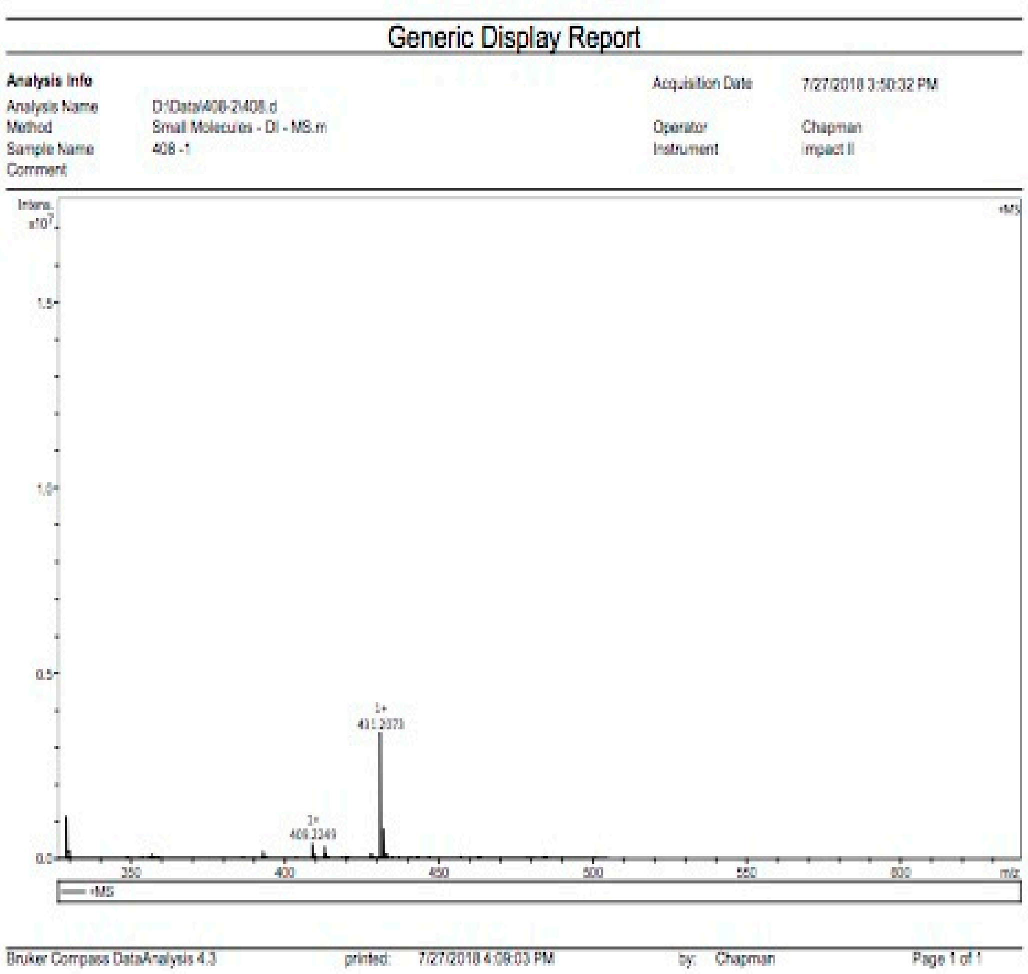

Figure S9. Mass spectra of (2E,2´E)-*N*,*N′*-(hexane-1,6-diyl) bis(3-(4-hydroxyphenyl) acrylamide) (**5**).

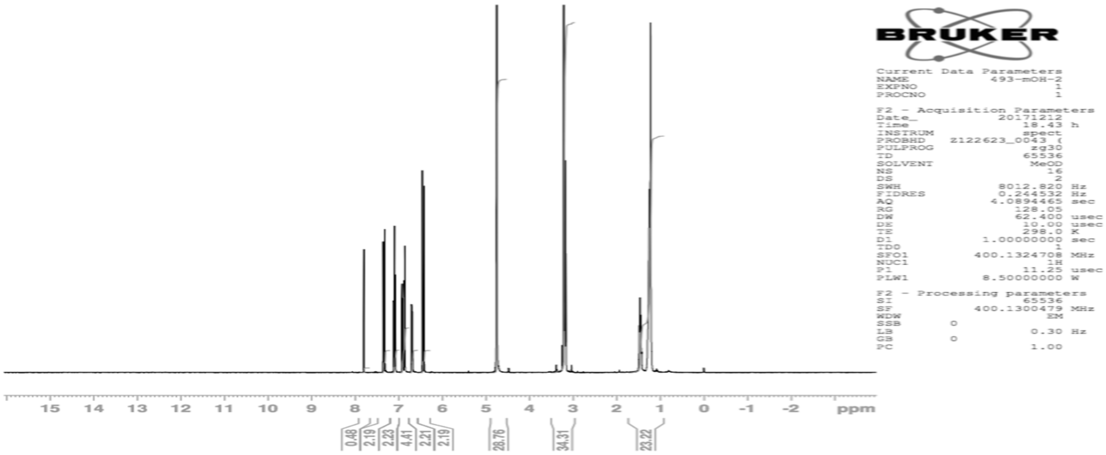

Figure S10. ^{1}H NMR (400 MHz, DMSO-d$_6$) of (2E,2´E)-*N*,*N′*-(dodecane-1,12-diyl)bis(3-(3 hydroxyphenyl)acrylamide) (**7**).

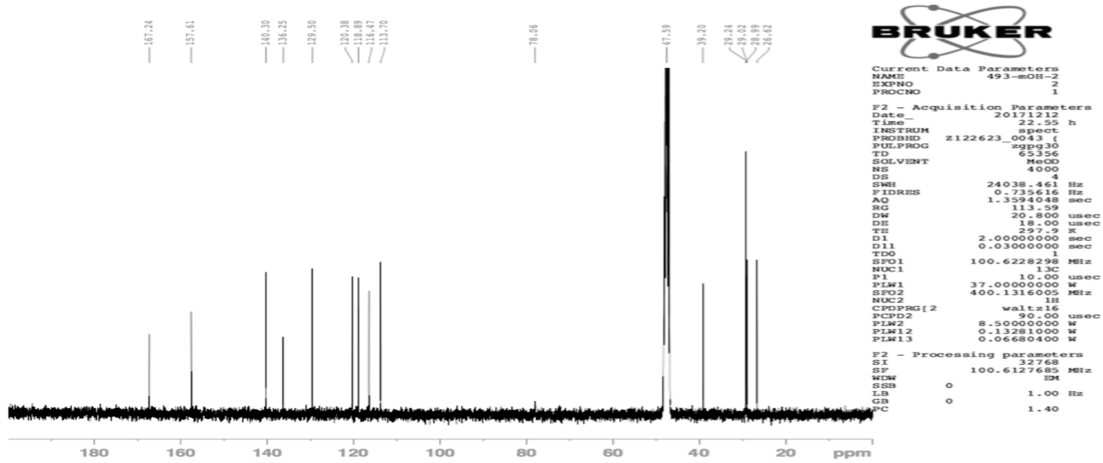

Figure S11. ^{13}C NMR (100 MHz, DMSO-d$_6$) of (2E,2´E)-*N*,*N′*-(dodecane-1,12-diyl) bis(3-(3-hydroxyphenyl) acrylamide) (**7**).

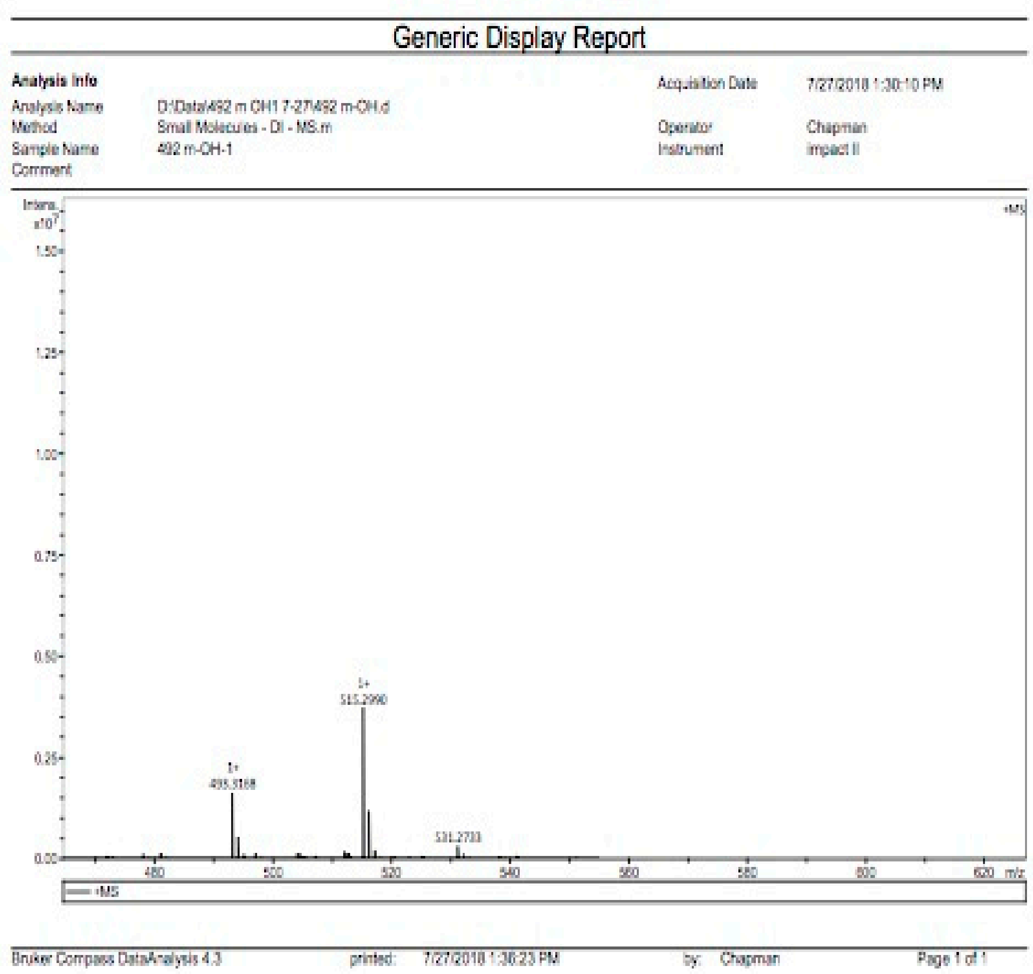

Figure S12. Mass spectra of (2E,2´E)-*N*,*N*′-(dodecane-1,12-diyl) bis(3-(3-hydroxyphenyl) acrylamide) (**7**).

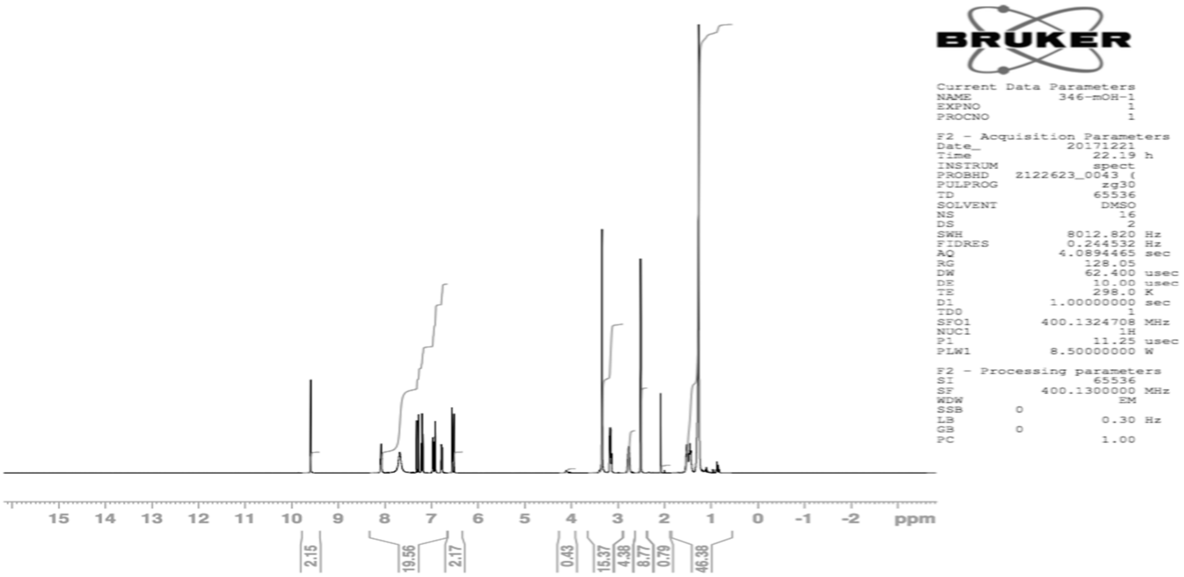

Figure S13. ^{1}H NMR (400 MHz, DMSO-d$_6$) of (E)-*N*-(12-aminododecyl)-3-(3-hydroxyphenyl) **(8)**.

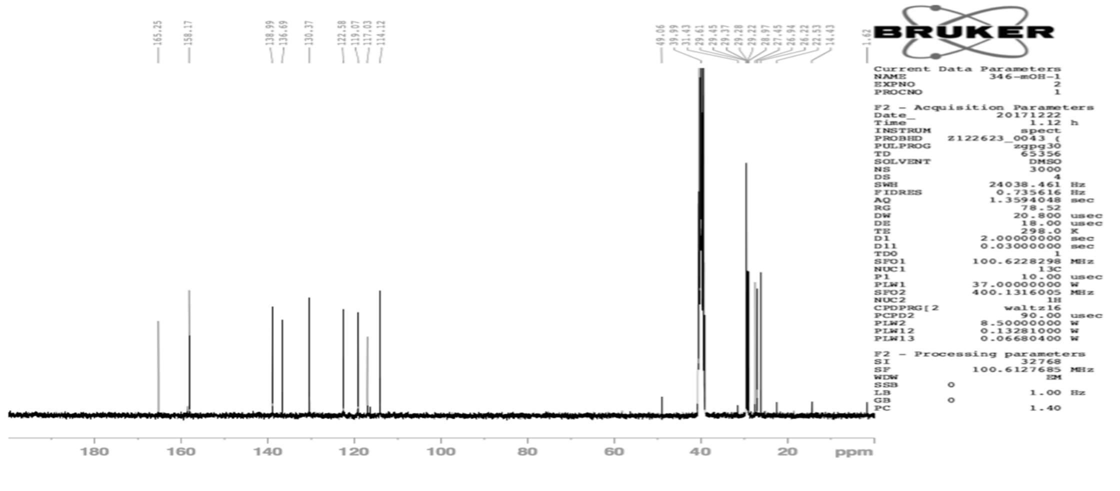

Figure S14. ^{13}C NMR (100 MHz, DMSO-d$_6$) of (E)-*N*-(12-aminododecyl)-3-(3-hydroxyphenyl) **(8)**.

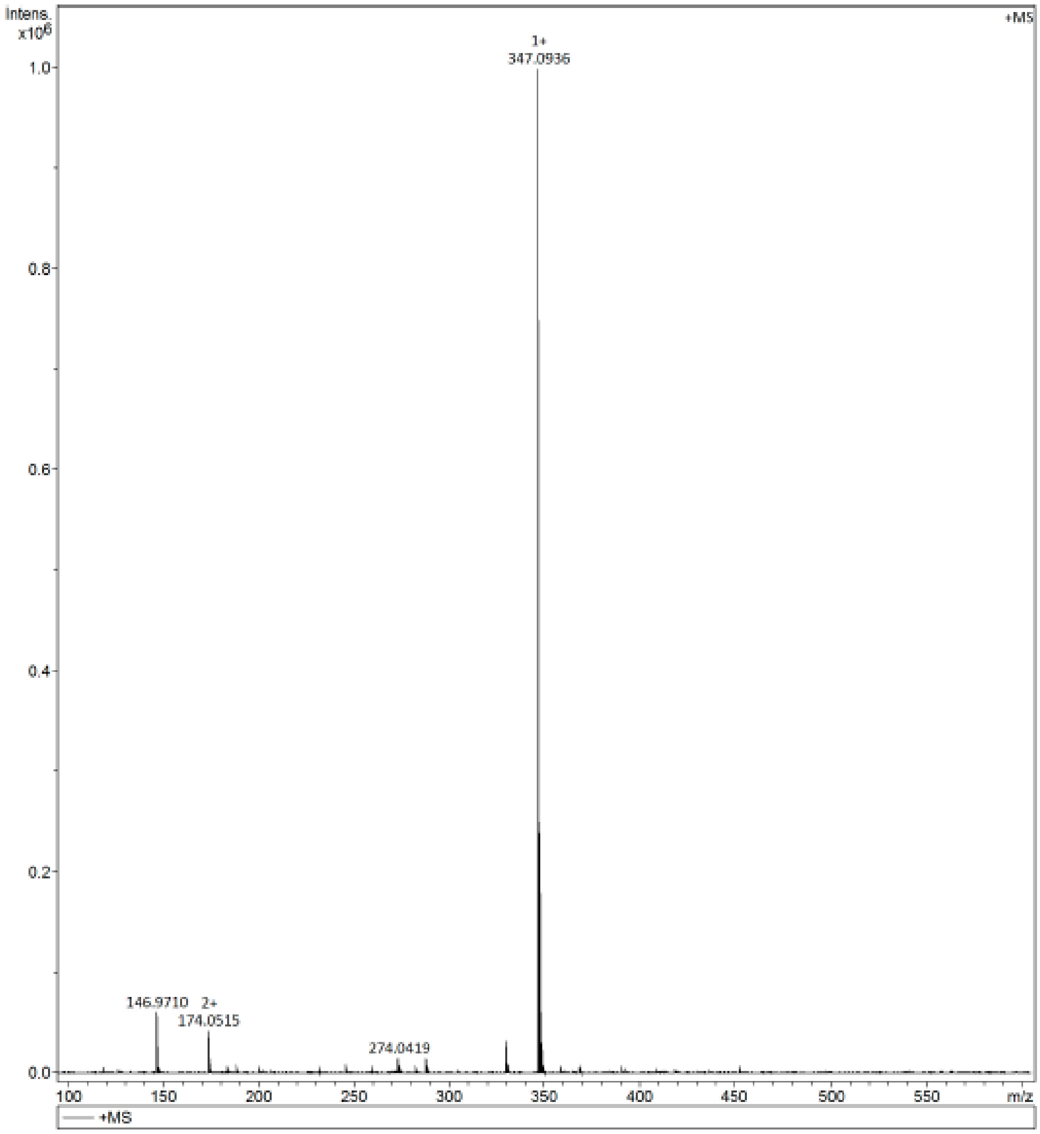

Figure S15. Mass spectra of (E)-*N*-(12-aminododecyl)-3-(3-hydroxyphenyl) **(8)**.

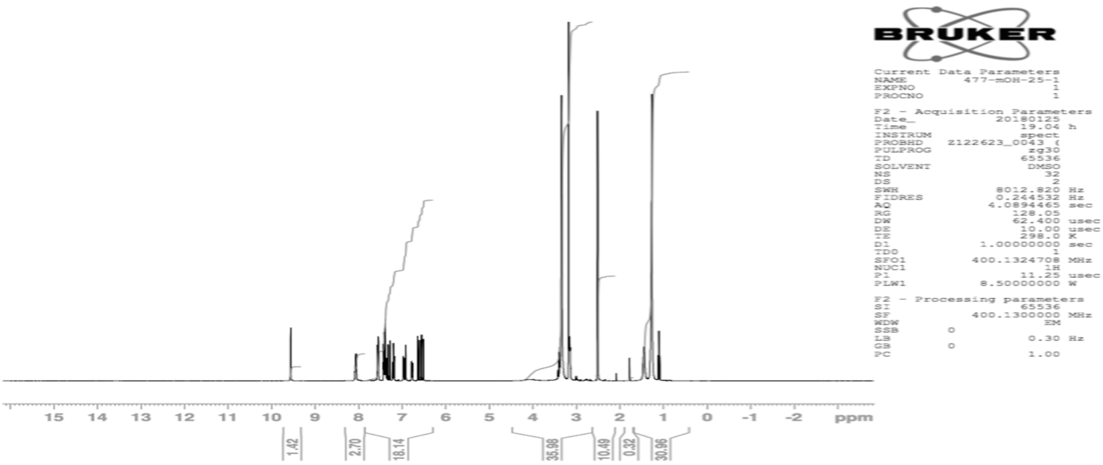

Figure S16. ^{1}H NMR (400 MHz, DMSO-d$_6$) of (E)-*N*-(12-cinnamamidododecyl)-3-(3-hydroxyphenyl) acrylamide (**9**).

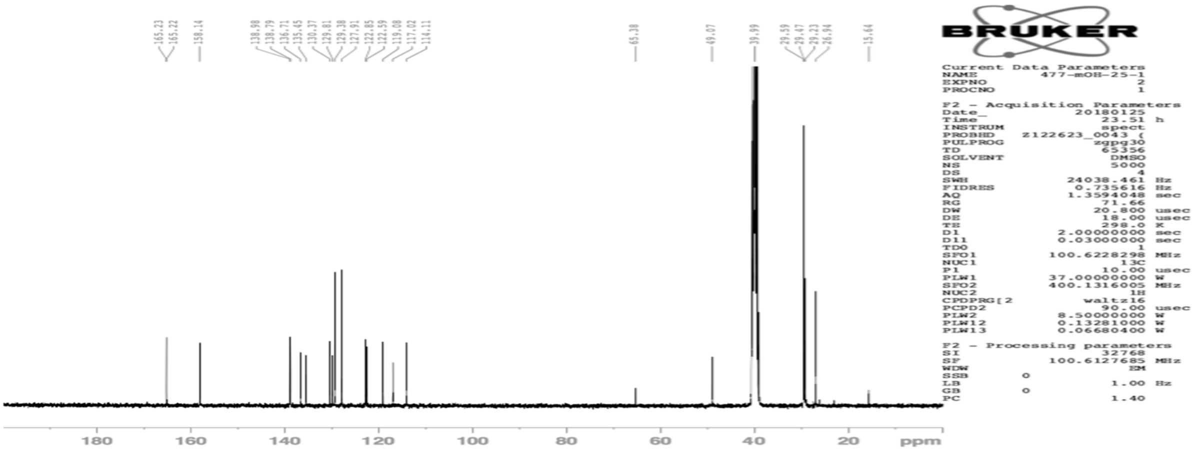

Figure S17. ^{13}C NMR (100 MHz, DMSO-d$_6$) of (E)-N-(12-cinnamamidododecyl)-3-(3-hydroxyphenyl) acrylamide (**9**).

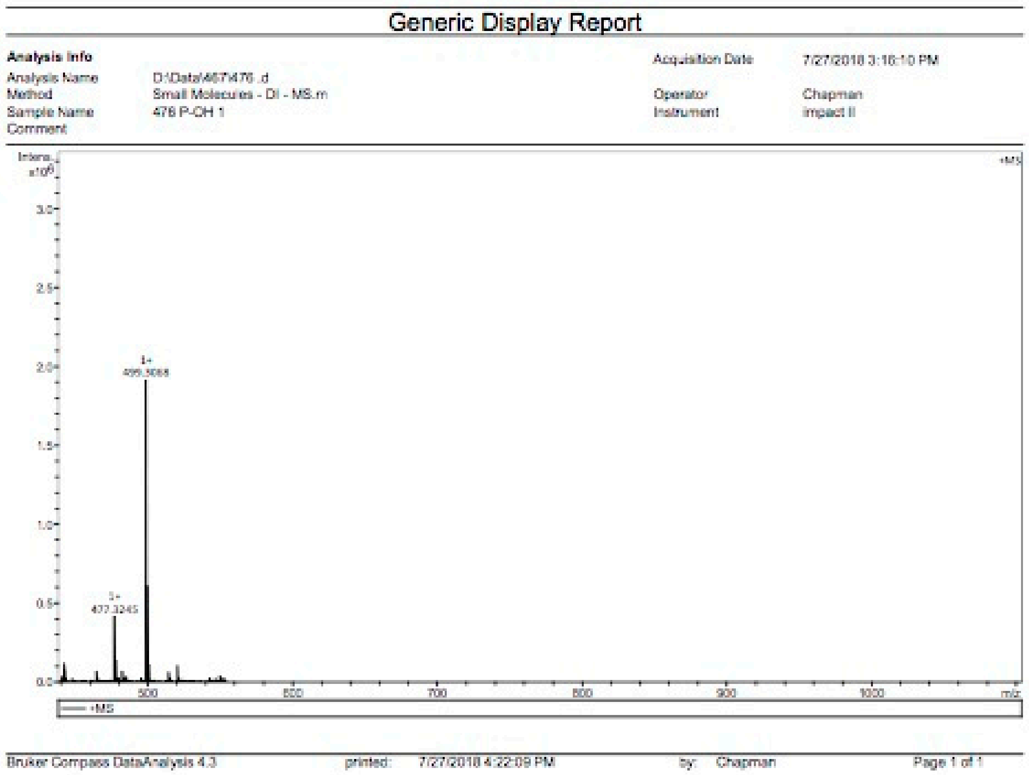

Figure S18. Mass spectra of (E)-*N*-(12-cinnamamidododecyl)-3-(3-hydroxyphenyl) acrylamide (**9**).

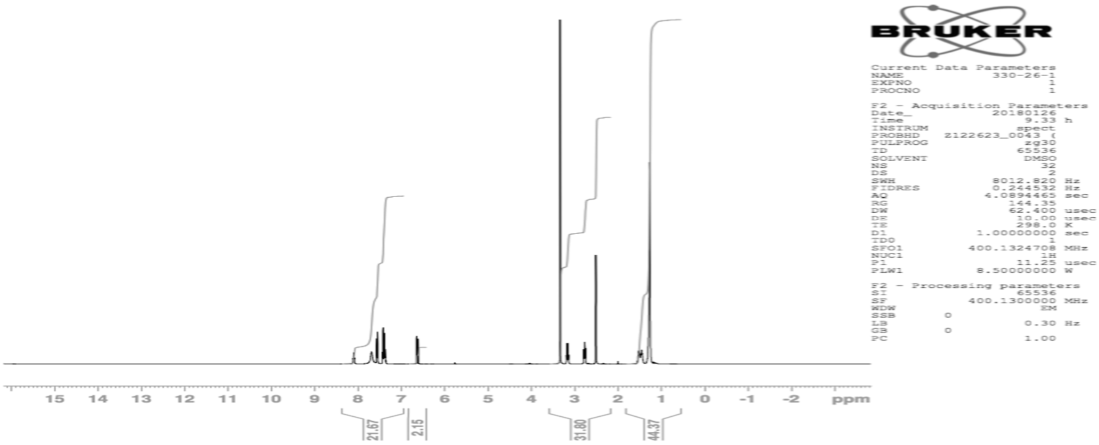

Figure S19. ^{1}H NMR (400 MHz, DMSO-d$_6$) of *N*-(12-aminododecyl) cinnamamide **(10)**.

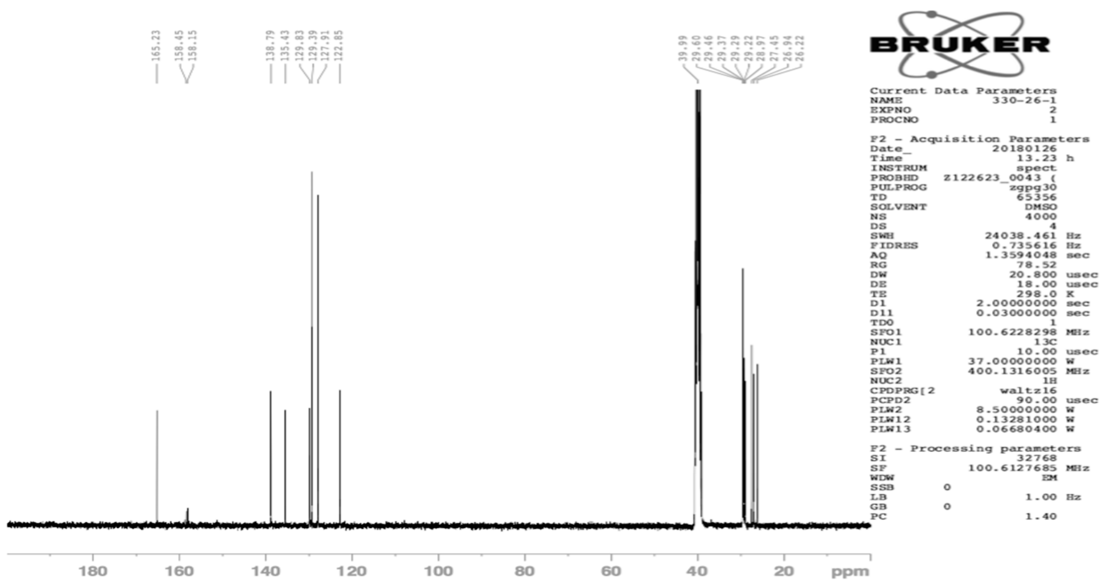

Figure S20: ^{13}C NMR (100 MHz, DMSO-d_6) of *N*-(12-aminododecyl) cinnamamide **(10)**.

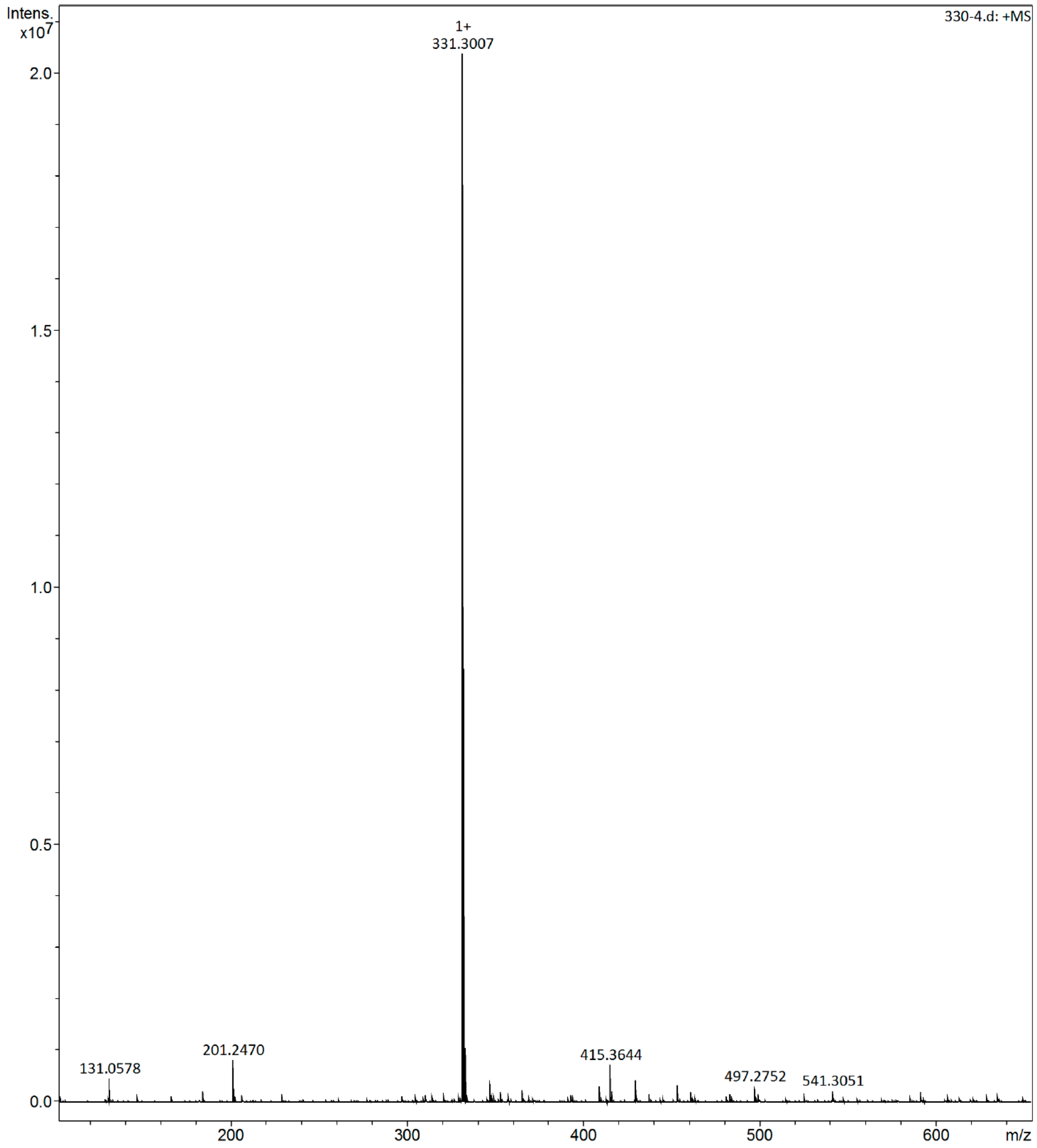

Figure S21. Mass spectra of N-(12-aminododecyl) cinnamamide (**10**).

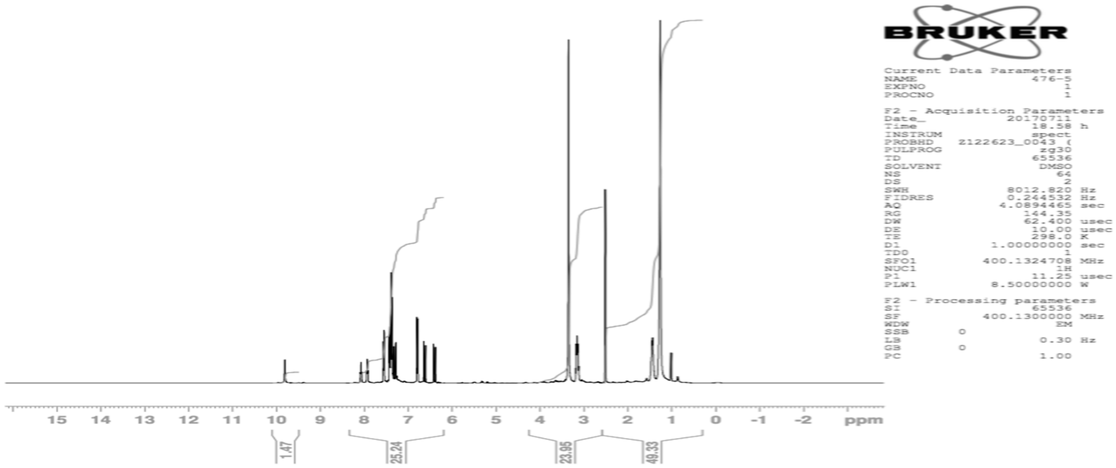

Figure S22. ^{1}H NMR (400 MHz, DMSO-d$_6$) of (E)-*N*-(12-cinnamamidododecyl)-3-(4-hydroxyphenyl) (**11**).

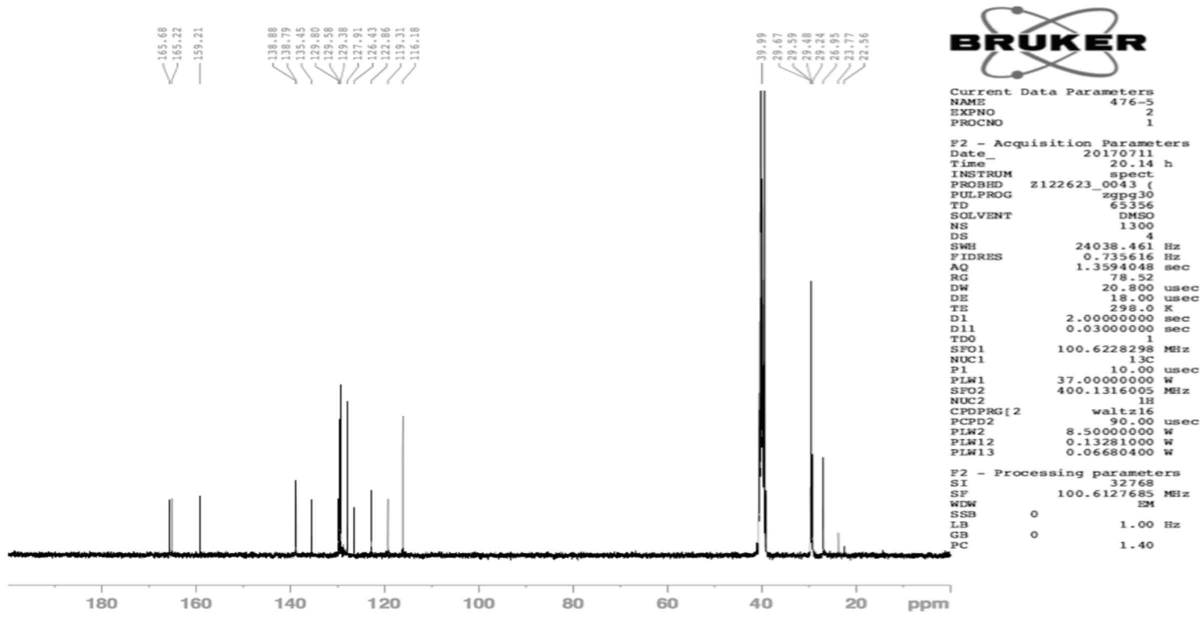

Figure S23. ^{13}C NMR (100 MHz, DMSO-d$_6$) of (E)-*N*-(12-cinnamamidododecyl)-3-(4-hydroxyphenyl) (**11**).

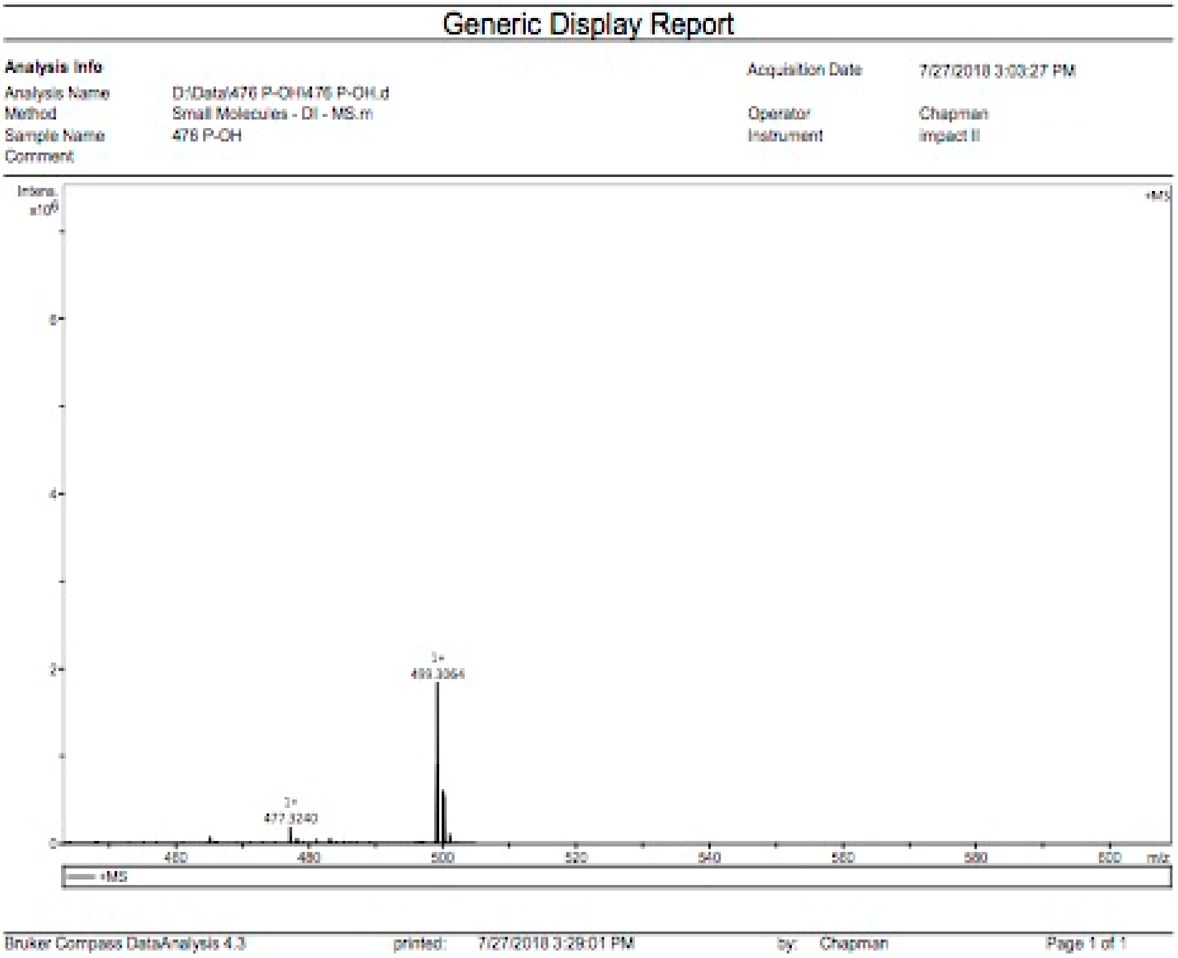

Figure S24. Mass spectra of (E)-*N*-(12-cinnamamidododecyl)-3-(4-hydroxyphenyl) (**11**).

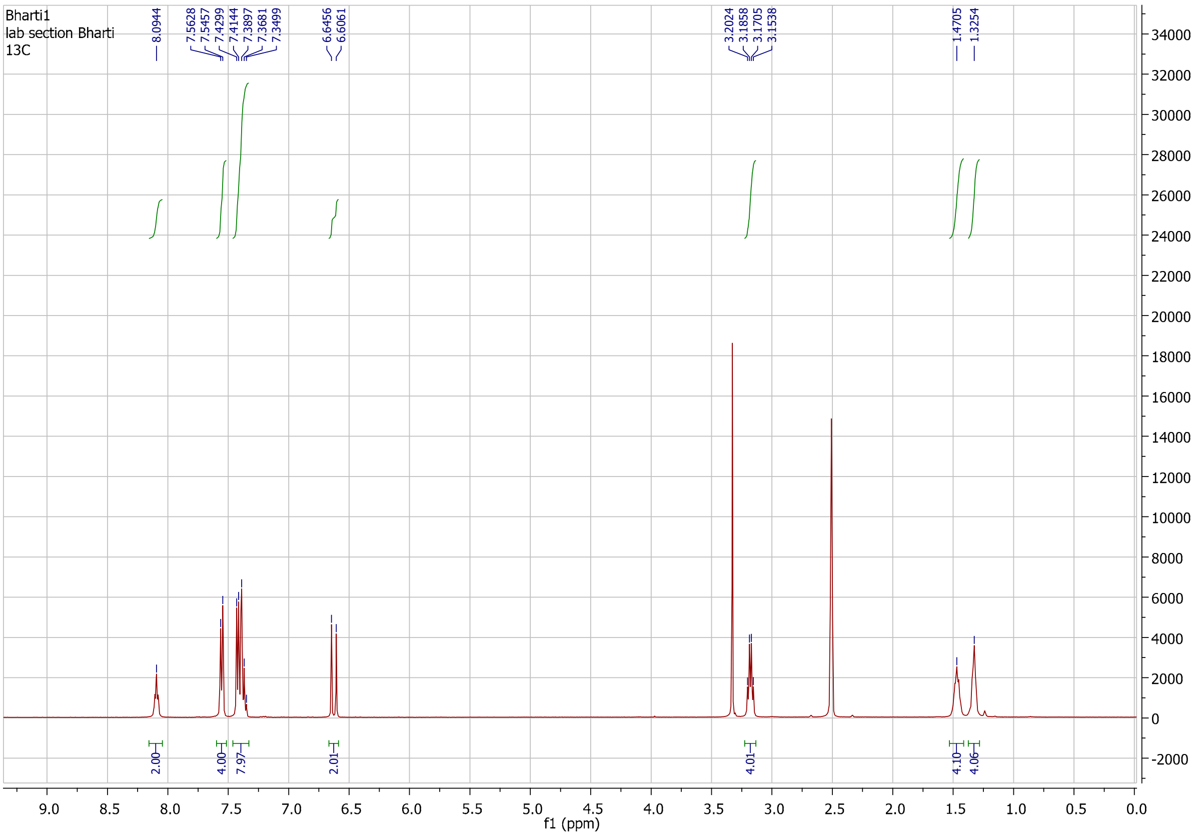

Figure S25. ^{1}H NMR (400 MHz, DMSO-d$_6$) of (2E,2'E)-*N*,*N*'-(hexane-1,6-diyl)bis(3-phenylacrylamide) **(12)**.

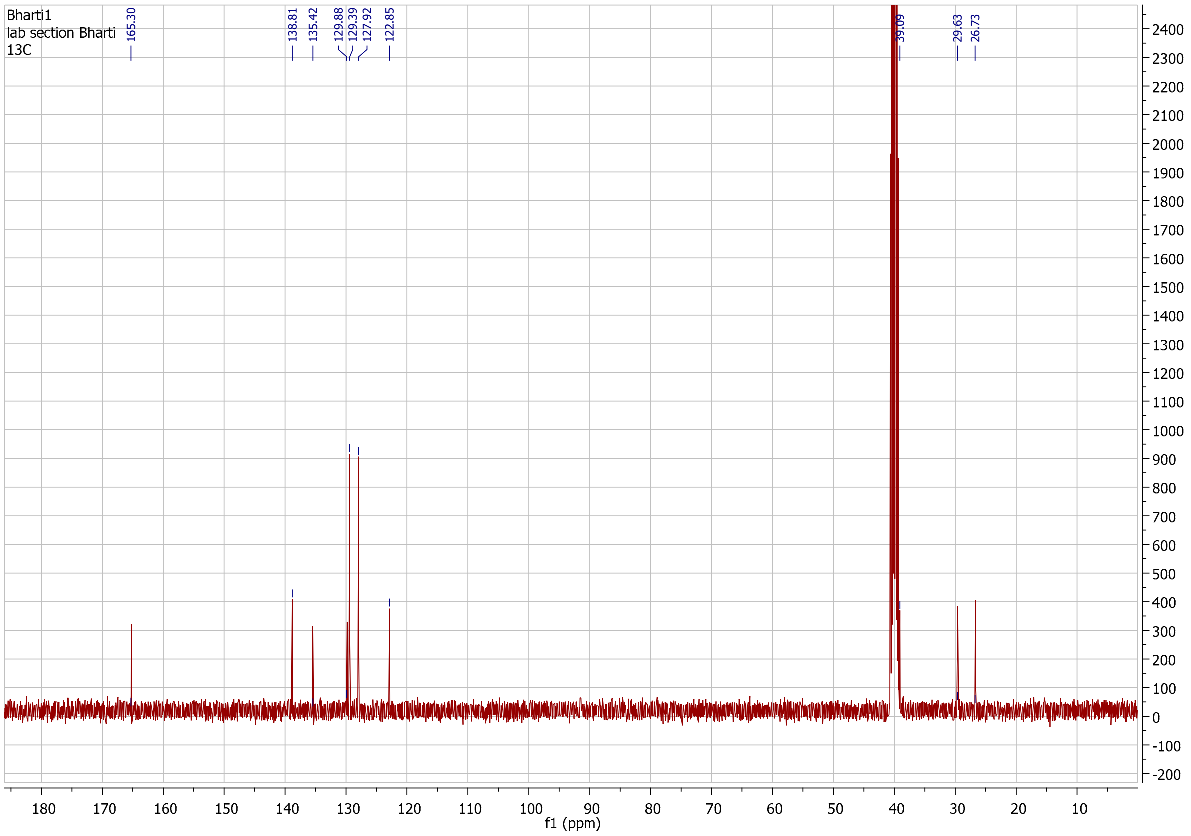

Figure S26. ^{13}C NMR (100 MHz, DMSO-d$_6$) of (2E,2'E)-*N*,*N'*-(hexane-1,6-diyl)bis(3-phenylacrylamide) **(12)**.

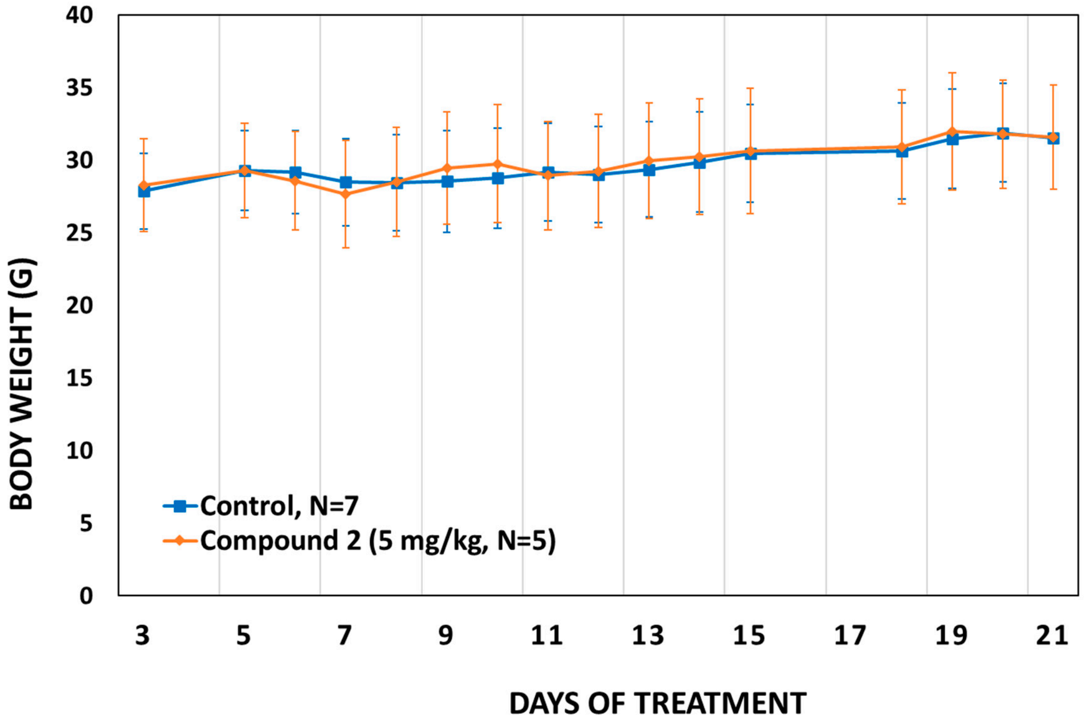

Figure S27. No significant bodyweight reduction was evident in mice treated with compound **2** (5 mg/kg, *i.p.*, daily) for 21 days in a melanoma xenograft mouse model. *P*>0.05 compared to control group.
